# Investigation of the free radical scavenging ability of l-tryptophan and its derivatives using experimental methods and quantum chemical calculations†

**DOI:** 10.1039/d4ra06729k

**Published:** 2024-11-29

**Authors:** Dinh Quy Huong, Pham Dinh Tu Tai, Nguyen Quang Trung, Nguyen Minh Thong, Nguyen Minh Tam, Nguyen Hai Phong, Pham Cam Nam

**Affiliations:** a Department of Chemistry, University of Education, Hue University Hue Vietnam dqhuong@hueuni.edu.vn; b Department of Planning, Finance and Facilities Management, Hue University Hue Vietnam; c Quality Assurance and Testing Center 2 Danang Vietnam; d The University of Danang, University of Science and Education Danang Vietnam; e Faculty of Basic Sciences, University of Phan Thiet 225 Nguyen Thong Phan Thiet City Binh Thuan Vietnam; f Department of Chemistry, University of Science, Hue University Hue Vietnam; g Department of Chemical Engineering, The University of Danang, University of Science and Technology Danang Vietnam

## Abstract

The free radical scavenging ability of l-tryptophan (LP) and 5-hydroxy-l-tryptophan (HLP) was evaluated using experimental and theoretical methods. The impact of antioxidant concentration on the scavenging of DPPH˙ and ABTS˙^+^ free radicals was assessed for both compounds. The results indicated that HLP exhibited superior scavenging ability, with IC_50_ values of 31.96 × 10^−7^ ± 0.85 × 10^−7^ M for DPPH˙ and 8.69 × 10^−6^ ± 0.95 × 10^−6^ M for ABTS˙^+^. In contrast, LP showed higher IC_50_ values of 9.51 × 10^−3^ ± 0.53 × 10^−3^ M for DPPH˙ assay and 8.91 × 10^−4^ ± 0.73 × 10^−4^ M for ABTS˙^+^ assay, indicating less effective scavenging. Theoretical calculations, performed by analyzing frontier molecular orbitals and molecular electrostatic potential, revealed that electron-donating regions were primarily distributed across the aromatic rings and heteroatoms. At the same time, electron-accepting zones were only located at nitrogen heteroatoms. The hydrogen atoms within the hydroxyl and amine groups of LP and HLP molecules were preferential positions for nucleophilic attacks. Furthermore, thermodynamic and kinetic analyses suggested that hydrogen atom transfer was the predominant mechanism governing the reaction of LP and HLP with free radicals. The presence of the OH group in the HLP molecule significantly enhanced its free radical scavenging ability compared to LP.

## Abstract 2

The presence of an OH group in 5-hydroxyl-l-tryptophan improves antioxidant ability.

## Graphical abstract

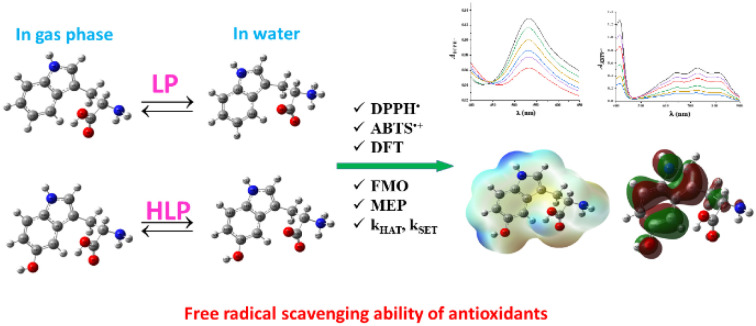

## 1. Introduction

Oxidation is a chemical process that generates free radicals – unstable and highly reactive molecules capable of causing cellular and tissue damage. Antioxidants are compounds that counteract these free radicals, thereby protecting cells from oxidative damage.^1^ They are utilized across various domains, including the protection of industrial materials, food preservation, and pharmaceutical applications.^2,3^

l-Tryptophan, known chemically as (2*S*)-2-amino-3-(1*H*-indol-3-yl)propanoic acid, represents an essential amino acid with multiple functions in human physiology. Present in various food sources such as milk, poultry, bread, chocolate, and bananas, l-tryptophan (LP) has been linked to enhanced nocturnal sleep, mood improvement in the elderly, and heightened levels of melatonin, serotonin, and total antioxidant capacity.^4^ The hydroxylated form of LP, 5-hydroxy-l-tryptophan (HLP), exhibits antioxidant properties and plays a pivotal role in melatonin synthesis and serotonin precursor function. Notably, non-toxic HLP is obtainable in substantial quantities from local sources such as cherries,^5^ coffee,^6^ tomatoes,^7^ and seeds of *Griffonia simplicifolia*^8^ or can be synthesized from LP.^9^ It has demonstrated efficacy in treating conditions like insomnia, cerebellar ataxia, chronic headaches, fibromyalgia, binge eating, and depression^10^ or serving as an antioxidant agent.^8^

A study investigating the hydrogen-donating ability of HLP through the 2,2-diphenyl-1-picrylhydrazyl experimental method unveiled its antioxidant virtues.^11^ Comparative assessments were conducted with mucuna seed (*Mucuna pruriens* var *utilis*) extract's antioxidant activities. In a study by C. Keithahn and A. Lerchl,^12^ HLP exhibited superior *in vitro* hydroxyl radical scavenging capabilities compared to melatonin or vitamin C, with a 50% inhibition concentration (IC_50_) of 1.8 μM.

While existing studies underscore the antioxidant potential of LP and HLP, the relationship between the structures of these substances in terms of antioxidant activity remains ambiguous. This study aims to evaluate the impact of structure and the presence of the hydroxyl group on the antioxidant capacity of LP and HLP using experimental methods and quantum chemical calculations.

The assessment will commence with evaluating free radical scavenging ability through the 2,2-diphenyl-1-picrylhydrazyl (DPPH˙) assay and 2,2′-azinobis(3-ethylbenzothiazoline-6-sulphonic acid) (ABTS˙^+^) assay. These methods involve scavenging the DPPH˙ free radical and neutralizing the ABTS˙^+^ free radical cation, primarily through the electron transfer process or hydrogen atom transfer mechanism.^13^ Subsequently, the theoretical approaches will scrutinize antioxidant activity, elucidating structural relationships influencing the compounds' antioxidant capacity.

The study will analyze factors including global descriptive parameters, molecular orbital energies, and charge distribution through the molecular electrostatic potential of investigated molecules to describe their reactivity.^14^ Thermodynamic parameters will be computed in the gas phase and water to simulate experimental conditions. Furthermore, the investigation will establish the potential energy surfaces of the reactions between LP and HLP with free radicals. The rate constants will be calculated from the various mechanisms to compare and select the optimal antioxidant compound.

## 2. Methods

### 2.1. Experimental methods

#### 2.1.1. Materials

**Fig. 1:**
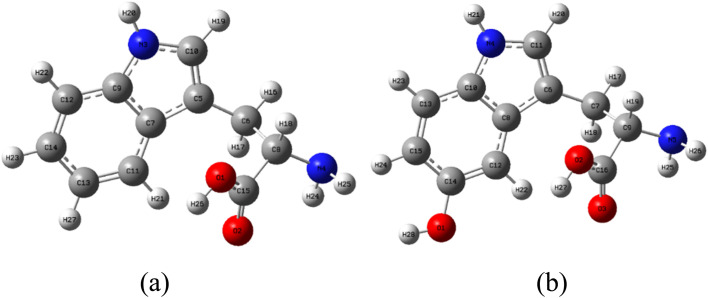
Stable structures of (a) LP and (b) HLP in the gas phase.

l-Tryptophan (C_11_H_12_N_2_O_2_) and 5-hydroxy-l-tryptophan (C_11_H_12_N_2_O_3_) were procured from Merk, Germany, and their structures are depicted in Fig. 1. The following reagents were sourced from Sigma-Aldrich: 2,2-diphenyl-1-picrylhydrazyl (DPPH˙), potassium persulfate (K_2_S_2_O_8_); 2,2′-azinobis(3-ethylbenzothiazoline-6-sulfonic acid) diammonium salt. Absolute ethanol was obtained from Fisher.

#### 2.1.2. 2,2-Diphenyl-1-picrylhydrazyl (DPPH˙) assay

A DPPH˙ solution was prepared in ethanol at a concentration of 6.7 × 10^−5^ M and protected from light by wrapping with aluminum foil to prevent photodegradation.^15^ The antioxidants under investigation, LP and HLP, were dissolved in water to a concentration range of 1 × 10^−3^ to 10 × 10^−3^ M for LP and 0.8 × 10^−6^ to 4.0 × 10^−6^ M for HLP. For each antioxidant concentration, 3 mL of the antioxidant solution was mixed with 1 mL of the prepared DPPH˙ solution, resulting in a reaction mixture. The mixture was thoroughly shaken and incubated in darkness for 30 minutes. Subsequently, the reaction mixture was transferred to a cuvette, and the absorbance was recorded at 515 nm using a SHIMADZU TCC-240A UV/vis spectrophotometer.

Based on the measured absorbance values, the DPPH˙ radical scavenging activity was calculated using eqn (1):^16^1
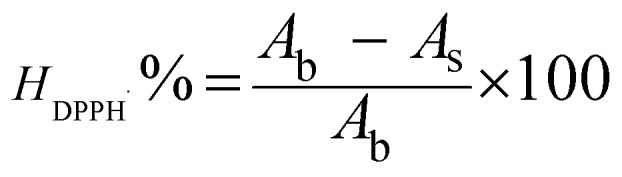
*H*_DPPH˙_%: DPPH˙ scavenging efficiency; *A*_b_: absorbance of the blank in the presence of DPPH˙. The blank solution consists of 1 mL of DPPH˙ and 3 mL of ethanol. *A*_s_: absorbance of the studied sample in the presence of DPPH˙. The sample solution comprises 1 mL of DPPH˙ and 3 mL of antioxidants at different concentrations.

Each experiment was repeated three times to ensure accuracy.

#### 2.1.3. 2,2′-Azinobis(3-ethylbenzothiazoline-6-sulfonate) (ABTS˙^+^) assay

The ABTS˙^+^ assay, a widely utilized method for assessing the antioxidant ability of substances, was conducted following the enhanced protocol of Re *et al.*^17^ Initially, the salt of ABTS˙^+^ radical cation, namely 2,2′-azinobis(3-ethylbenzothiazoline-6-sulfonic acid) diammonium, was dissolved in deionized water to a concentration of 7 mM. This solution was mixed with 140 mM potassium persulfate (K_2_S_2_O_8_) and held in the dark at room temperature for 16 hours to generate the ABTS˙^+^ radical cation. The formation of ABTS˙^+^ was indicated by the development of a blue-green color.^18^

Before use, the ABTS˙^+^ solution was diluted with ethanol to absorb approximately 0.7 at 734 nm. Antioxidants were then appropriately diluted in water, with concentrations ranging from 8 × 10^−4^ to 10^−2^ M for LP and from 0.8 × 10^−6^ to 4.0 × 10^−6^ M for HLP. A fixed volume of the diluted ABTS˙^+^ solution (3 mL) was combined with a volume of the antioxidant sample solution (1 mL), and this mixture was incubated in the dark for 6 minutes.

Following incubation, the reaction mixture was transferred to a cuvette, and its absorbance was measured at 734 nm using a TCC-240A SHIMADZU UV/vis spectrophotometer. All experiments were conducted in triplicate, and similar procedures were followed for the different samples.

The ABTS˙^+^ radical cation capturing ability was calculated from the absorbance using eqn (2).^19^2
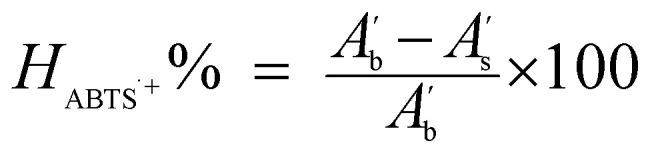
*H*_ABTS˙^+^_%: ABTS˙^+^ scavenging efficiency; 
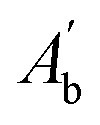
: absorbance of the blank in the presence of ABTS˙^+^. The blank consisted of 3 mL of ABTS˙^+^ mixed with 1 mL of ethanol. 
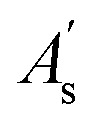
: absorbance of the sample in the presence of ABTS˙^+^. The sample consisted of 3 mL of ABTS˙^+^ and 1 mL of antioxidants at different concentrations.

### 2.2. Theoretical approaches

Density functional theory (DFT) calculations were performed using Gaussian 16 software to investigate the studied compounds.^20^ Initial geometry optimizations were carried out at the M06-2X/6-311++G(d,p) level of theory to identify the most stable configurations of LP and HLP.^21^ Subsequently, global descriptive parameters of selected molecules were calculated with the following equations:^22,23^

3 Δ*E*_L–H_ = *E*_LUMO_ − *E*_HOMO_

4 IE = −*E*_HOMO_

5 EA = −*E*_LUMO_

6
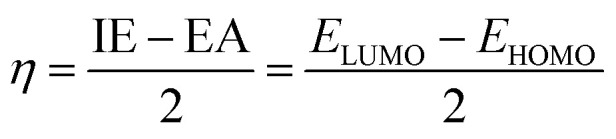
7
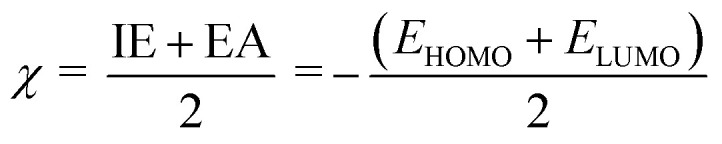


In which, *E*_HOMO_ and *E*_LUMO_ are the highest occupied molecular orbital energy and the lowest unoccupied molecular orbital energy. Δ*E*_L–H_ denotes the frontier molecular orbital gap, *η* and *χ* are hardness and calculated electronegativity of the studied compound. These parameters will provide information about the stability and reactivity of the molecule at the molecular level.

The antioxidant mechanisms of LP and HLP were investigated systematically in both gas and aqueous phases. The effect of water as a solvent was estimated using the IEFPCM solvation model.^24^ The studied antioxidant mechanisms included hydrogen atom transfer (HAT), single-electron transfer followed by proton transfer (SELPT), and sequential proton loss electron transfer (SPLET). The HAT mechanism relates to N–H and O–H bonds' bond dissociation enthalpy (BDE).^25^ SELPT mechanism focuses on the ionization potential (IP) and proton dissociation enthalpy (PDE),^26,27^ while the proton affinity (PA) and electron transfer enthalpy (ETE) are critical quantities for the SPLET mechanism.^28,29^ Detailed calculations of all the aforementioned thermodynamic parameters were presented in Table S1 of the ESI† data file.

Additionally, the reactivity between antioxidants and free radicals is affected by their rate constants (*k*). According to Marcus theory,^30,31^ the single electron transfer (SET) reaction relies on the transition state to determine the electron transfer activation barrier (Δ*G*^≠^_ET_) as follows:^32^8
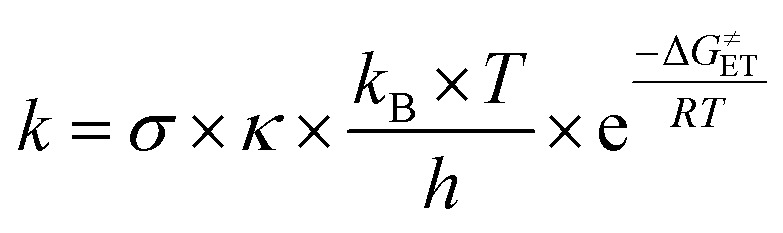
where *σ* is the total symmetry number of the reaction, *k*_B_ is Boltzmann's constant, *κ* denotes a tunneling correction factor, *R* is the gas constant, *T* is the temperature in Kelvin, and *h* is Planck's constant.

The electron transfer activation barrier (Δ*G*^≠^_ET_) is calculated using:9
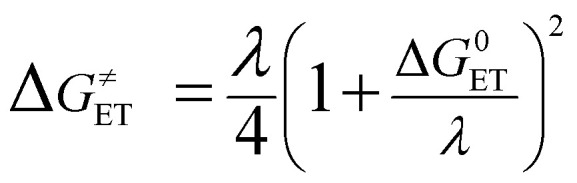
where the Δ*G*^0^_ET_ is the free energy of the reaction and *λ* denotes the nuclear reorganization energy, determined by:

10 *λ* ≈ Δ*E*_ET_ − Δ*G*^0^_ET_

where Δ*E*_ET_ is the non-adiabatic energy difference between reactants and vertical products.

The rate constant for the HAT mechanism is calculated at M06-2X/6-311++G(d,p) using the Eyringpy software.^33^ While various pathways can be considered, it is widely accepted that the antioxidant activity of both LP and HLP should be assessed through two primary mechanisms: HAT and SET. The overall rate constant (*k*_tot_) is determined as described in:^34^

11 *k*_tot_ = *k*_HAT_ + *k*_SET_

The individual HAT rate constants, *k*_HAT(i)_, sum up to give the total HAT rate constant, *k*_HAT_. Likewise, the individual SET rate constants, *k*_SET(i)_, sum up to yield the total SET rate constant, *k*_SET_.

12 *k*_HAT_ = Σ*k*_HAT(i)_

13 *k*_SET_ = Σ*k*_SET(i)_

The proportions of products (*P*%) formed through different reaction mechanisms can be calculated using these equations.14
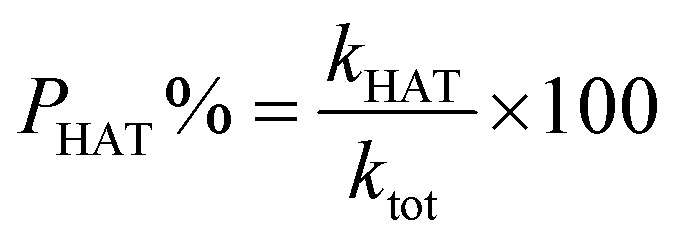
15
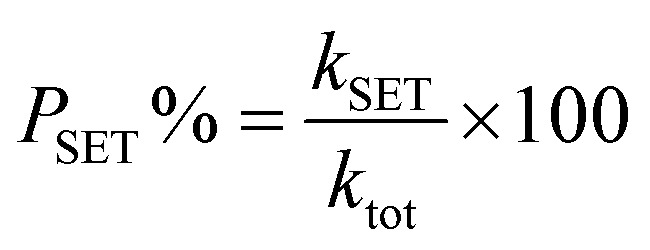
where *P*_HAT_% and *P*_SET_% are the proportions of products formed through HAT and SET mechanisms.

## 3. Results and discussion

### 3.1. Experimental results

The 2,2-diphenyl-1-picrylhydrazyl (DPPH˙) assay is used to evaluate the antioxidant capacity of various compounds. The assay relies on the reduction of the stable DPPH˙ radical, characterized by its purple color, to the yellow-colored diphenylpicrylhydrazine upon interaction with an antioxidant capable of donating a hydrogen atom.^35^ The extent of DPPH˙ reduction, indicative of antioxidant activity, is quantified by measuring the decrease in absorbance at 515 nm.

**Fig. 2:**
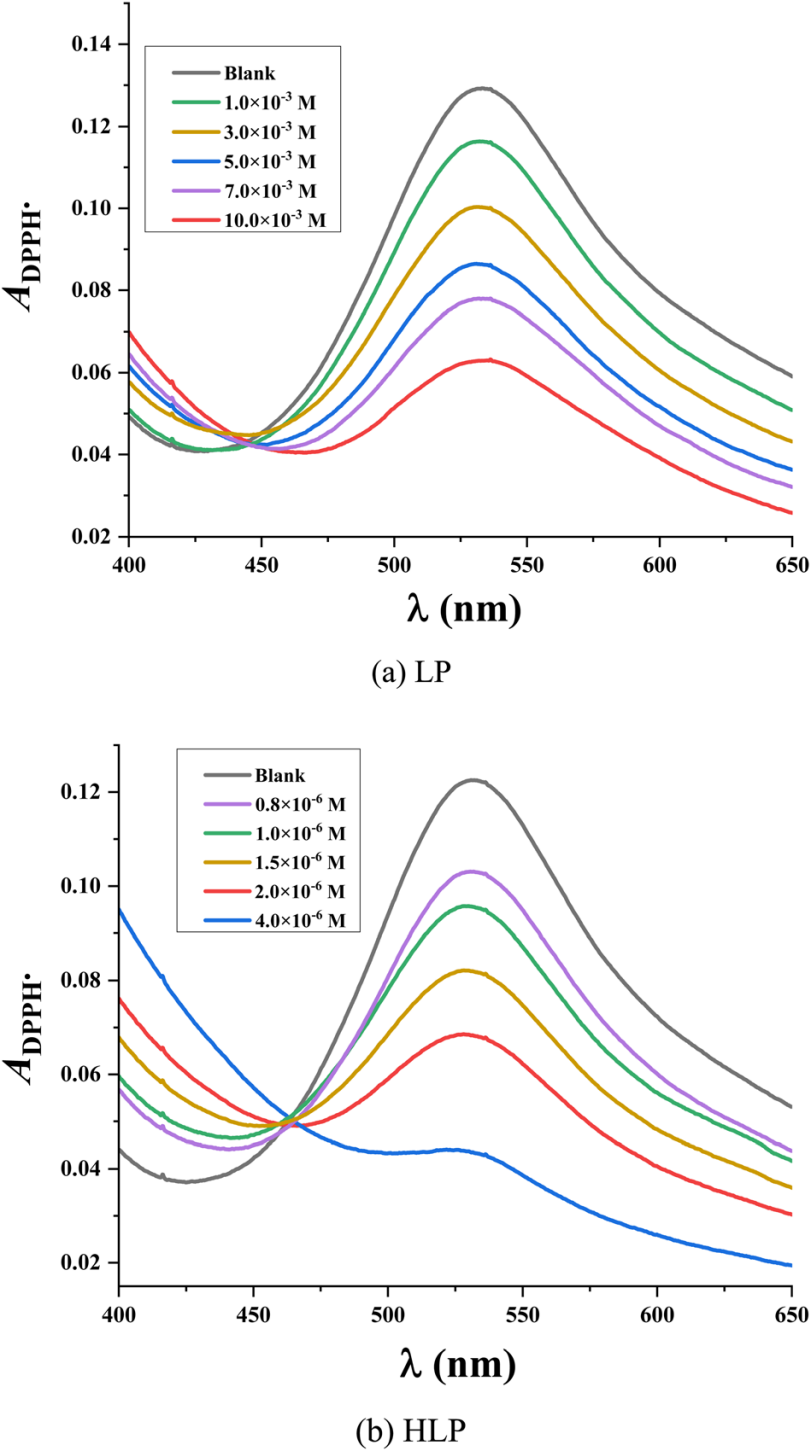
UV-vis spectra of (a) LP and (b) HLP in the investigation of DPPH˙ experiments.

**Fig. 3:**
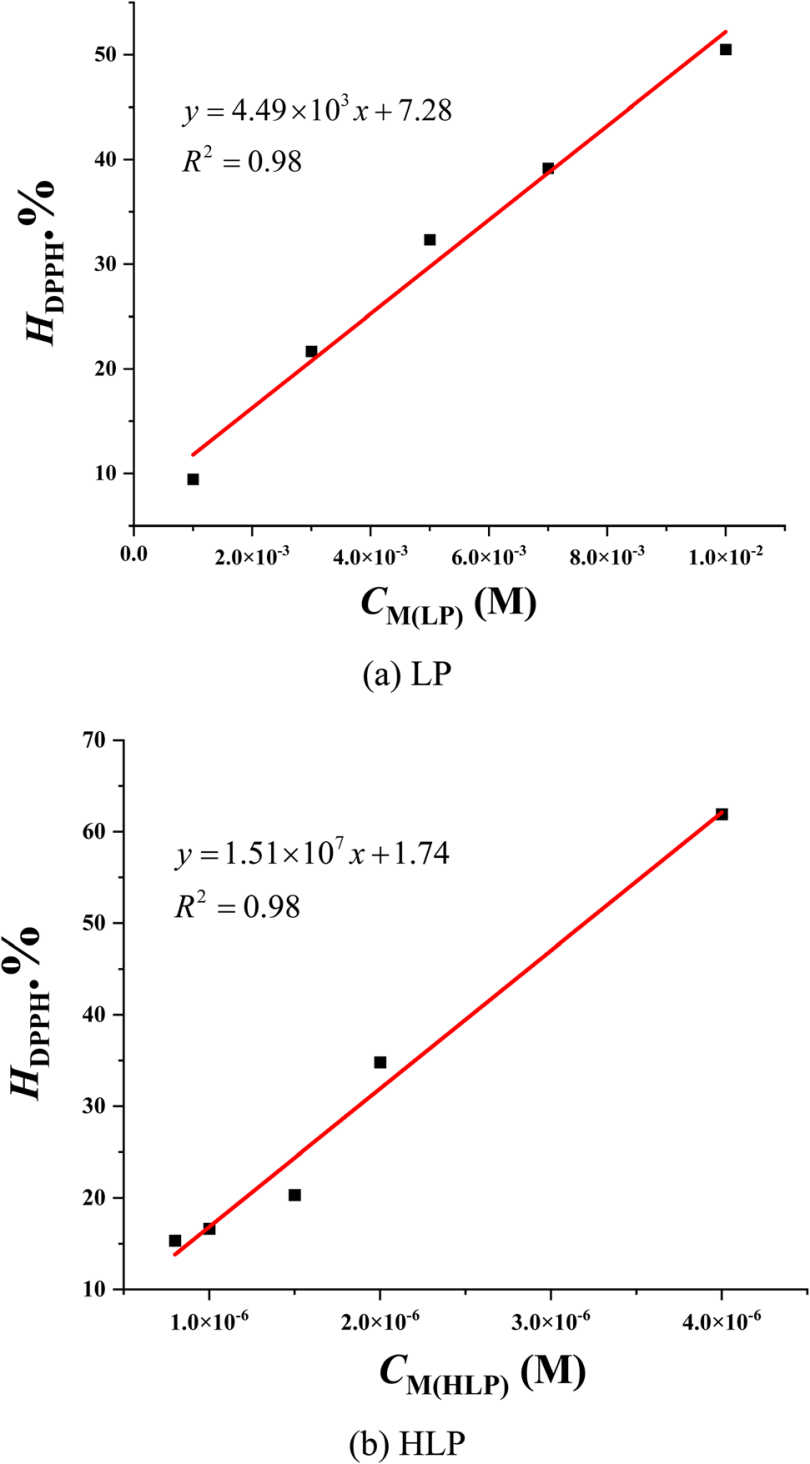
Correlation between the concentration of (a) LP, (b) HLP and their DPPH˙ scavenging efficiency.

As depicted in Fig. 2, the decrease in optical density of the DPPH˙ solution upon increasing concentrations of LP and HLP indicates a concentration-dependent scavenging capacity. The IC_50DPPH_ value, which shows the concentration required to inhibit 50% of the initial DPPH˙ radicals, is determined to evaluate the free radical capturing abilities of LP and HLP. The IC_50DPPH_ values are found to be 9.51 × 10^−3^ ± 0.53 × 10^−3^ M and 31.96 × 10^−7^ ± 0.85 × 10^−7^ M for LP and HLP (Fig. 3). These results demonstrate that HLP exhibits significantly stronger DPPH˙ scavenging activity than LP.

**Fig. 4:**
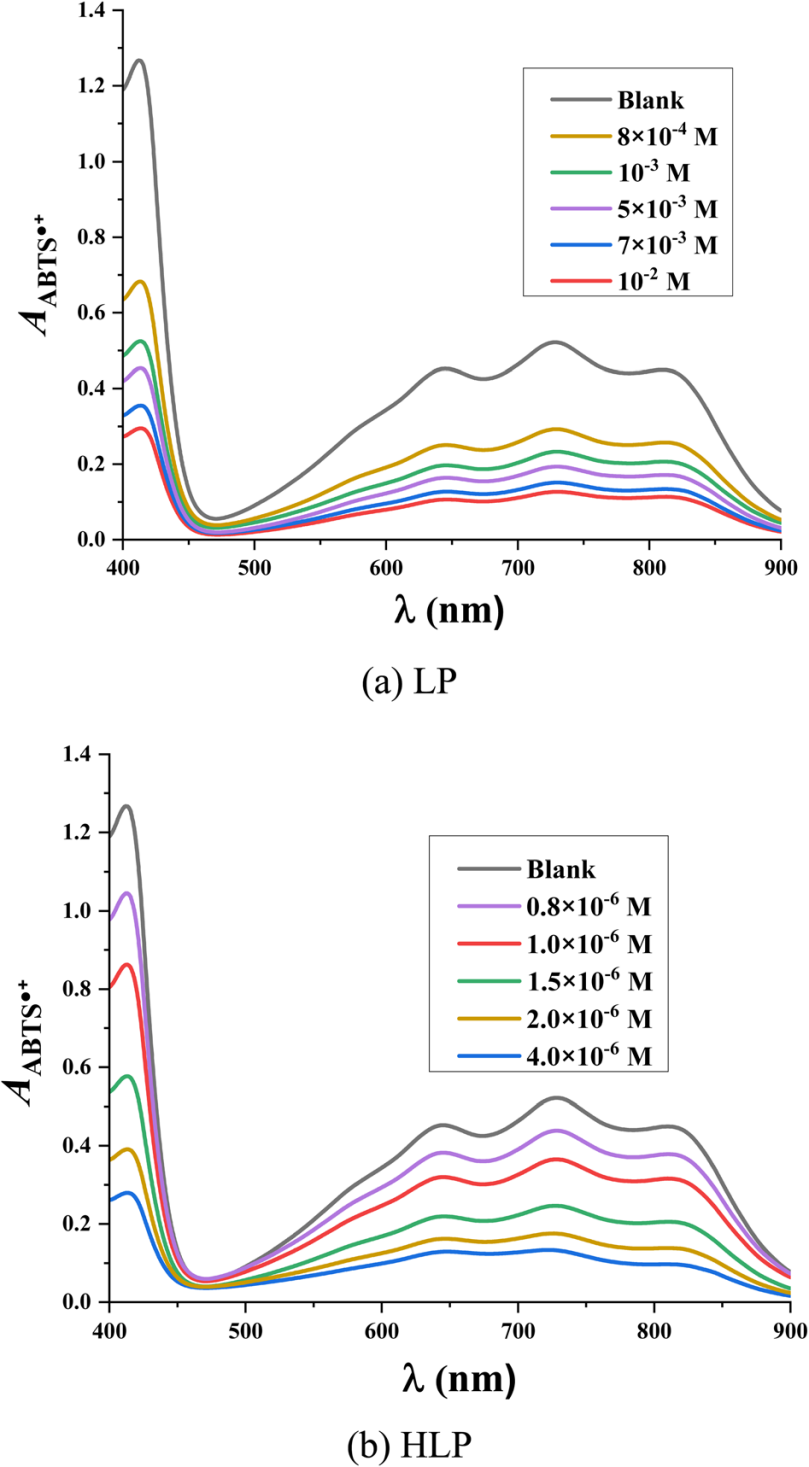
UV-vis spectra of (a) LP and (b) HLP in the investigation of ABTS˙^+^ experiments.

The antioxidant capacity of the studied derivatives is assessed using the ABTS˙^+^ assay. In this assay, the radical cation ABTS˙^+^ forms a blue-green colored solution with a maximum absorbance at 734 nm. Upon reaction with antioxidants, the blue color is decolorized.^36^Fig. 4 illustrates the ultraviolet-visible spectra of LP and HLP in the ABTS˙^+^ assay. The spectral changes observed in the ABTS˙^+^ test follow a similar trend to those in the DPPH˙ test. With increasing concentrations of LP and HLP, the absorbance of the solution decreases, indicating a reduction in the concentration of ABTS˙^+^ radical cations in the solution.

**Fig. 5:**
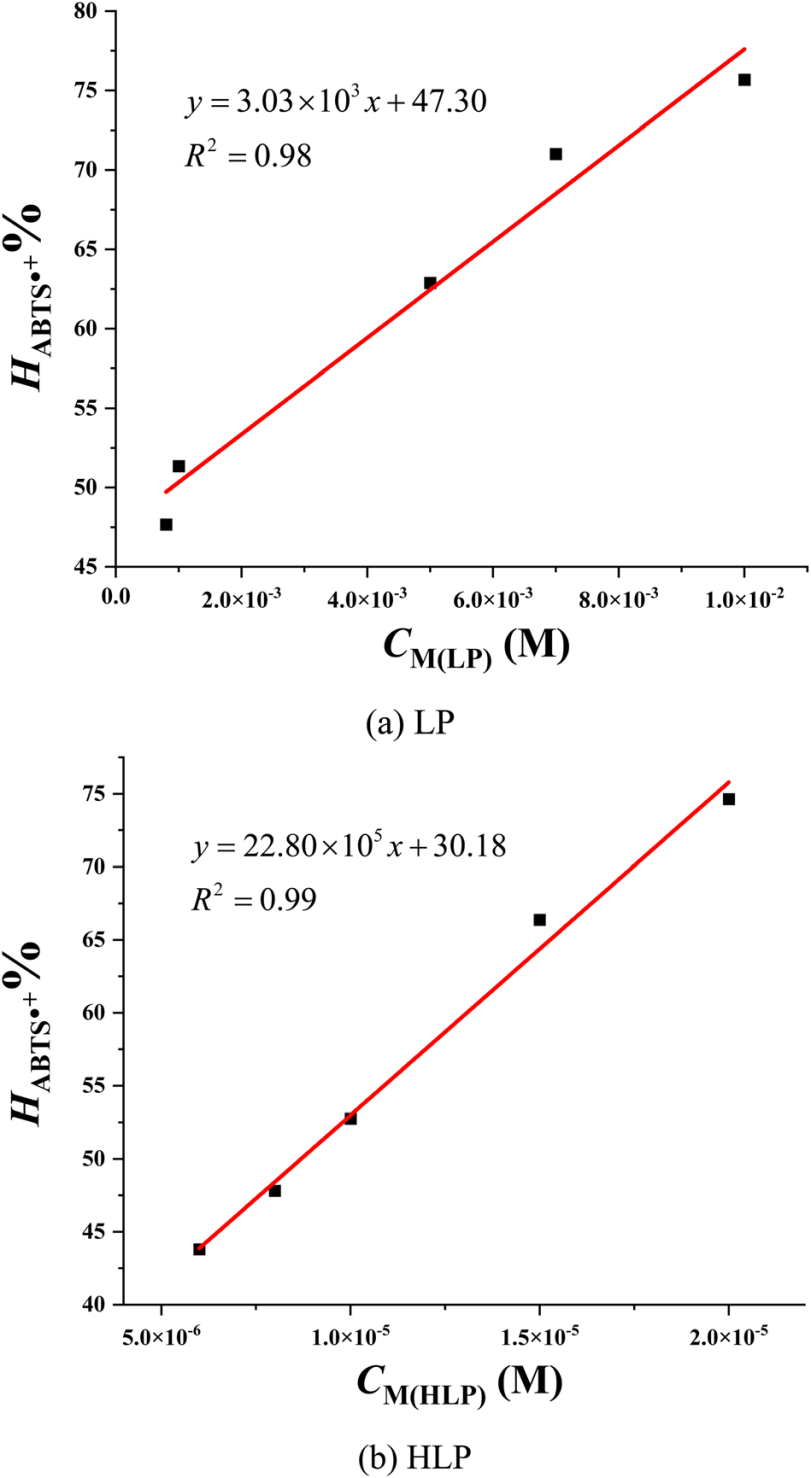
Correlation between the concentration of (a) LP, (b) HLP and their ABTS˙^+^ scavenging efficiency.

The IC_50ABTS_ values for LP and HLP are determined based on the relationship between the ABTS˙^+^ radical cation scavenging ability (*H*_ABTS˙^+^_%) and the concentration of antioxidants, as depicted in Fig. 5. LP has an IC_50ABTS_ value of 8.91 × 10^−4^ ± 0.73 ×10^−4^ M, while HLP has a value of 8.69 × 10^−6^ ± 0.95 × 10^−6^ M. These results indicate that HLP is more effective at neutralizing ABTS˙^+^ radical cations than LP. Both experimental methods, DPPH˙ and ABTS˙^+^, demonstrate that HLP is more effective at quenching free radicals than LP.

### 3.2. Computational results

Before further analysis, the structures of LP and HLP are optimized using the M06-2X/6-311++G(d,p) level of theory. The corresponding Cartesian coordinates are provided in the supplementary data (Tables S2–S6†). Due to the functional groups' rotational flexibility, multiple LP and HLP conformations exist. Only the most stable conformations are selected for analysis to ensure the accuracy of the theoretical calculations. Water is chosen as the solvent to mimic experimental conditions and provide a more realistic representation of the molecules' behavior in a biological environment.

#### 3.2.1. Frontier molecular orbitals and reactivity descriptors

**Fig. 6:**
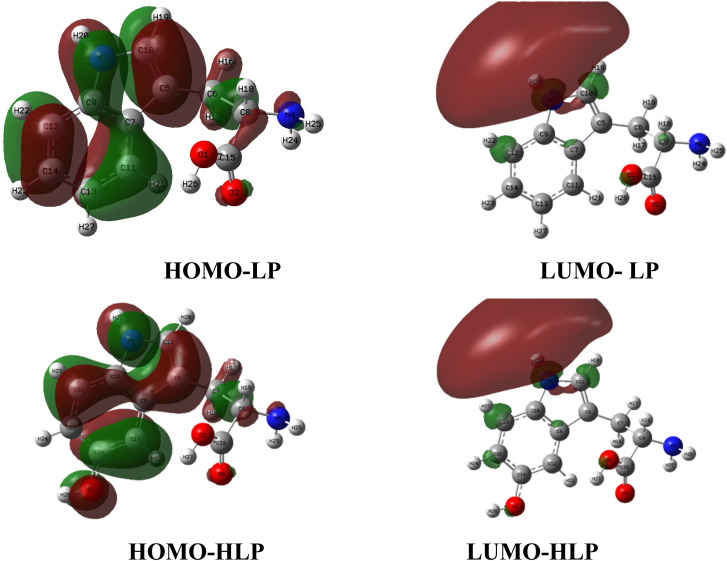
HOMO and LUMO plots of LP and HLP in the gas phase.

Frontier molecular orbitals of LP and HLP are analyzed to obtain a deeper understanding of their chemical reactivity of LP and HLP. The highest occupied molecular orbital (HOMO) and the lowest unoccupied molecular orbital (LUMO) of investigated compounds are depicted in Fig. 6.^37,38^ While HOMO provides information on electron-donating regions, LUMO reveals the electron-accepting sites. For both LP and HLP, the electron donor regions are distributed throughout the aromatic rings and heteroatoms, whereas the electron acceptor regions are only concentrated at the nitrogen heteroatoms.

Numerous studies indicate that quantum chemical parameters can describe the stability and reactivity of a compound at the molecular level.^39,40^ In particular, *E*_HOMO_ is directly related to free radical scavenging activity.^41^ Therefore, the *E*_HOMO_ values of LP and HLP are calculated and presented in Table 1. The *E*_HOMO_ value of HLP is −6.89 eV, which is significantly higher than that of LP (−7.02 eV). This indicates that HLP is more likely to scavenge free radicals compared to LP.

**Table 1:** Molecular parameters of LP and HLP at the M06-2X/6-311++G(d,p)

Compounds	LP		HLP	
	Gas	Water	Gas	Water
*E*_HOMO_ (eV)	−7.02	−7.01	−6.89	−6.93
*E*_LUMO_ (eV)	−0.26	0.02	−0.28	0.02
Δ*E*_LUMO–HOMO_ (eV)	6.76	7.03	6.61	6.95
*η* (eV)	3.38	3.52	3.30	3.48
*χ* (eV)	3.64	3.50	3.59	3.46

Furthermore, the distance between the frontier molecular orbitals is an important parameter. A smaller Δ*E*_LUMO–HOMO_ indicates greater polarity.^34^ As shown in Table 1, in the gas phase, HLP has a Δ*E*_LUMO–HOMO_ of 6.61 eV, while for LP, it is 6.76 eV. A similar trend is observed in water, where HLP has a Δ*E*_LUMO–HOMO_ of 6.95 eV, and LP has 7.03 eV. This indicates that HLP is more polar than LP, confirming its higher reactivity with free radicals.^42^

Additionally, global descriptive parameters such as hardness (*η*) provide valuable insights into the reactivity of the investigated compounds.^43^ Hardness (*η*) is directly related to molecular stability, a higher hardness value indicates greater resistance to reaction. In this study, HLP exhibits lower hardness values of 3.30 eV in the gas phase and 3.48 eV in water, suggesting it is more reactive towards free radicals than LP.

Next, electronegativity (*χ*) should also be considered to gain deeper insight into charge–transfer reactions.^44^ A lower electronegativity suggests a higher propensity for a molecule to donate electrons, contributing to antioxidant activity. Compared to the LP, HLP exhibits lower electronegativity values in the gas phase (3.59 eV) and water (3.46 eV), highlighting its stronger electron-donating ability.

Overall, the global reactivity descriptor values suggest that HLP has a greater potential for antioxidant activity than LP, primarily through the electron scavenging reactions.

#### 3.2.2. Molecular electrostatic potential

**Fig. 7:**
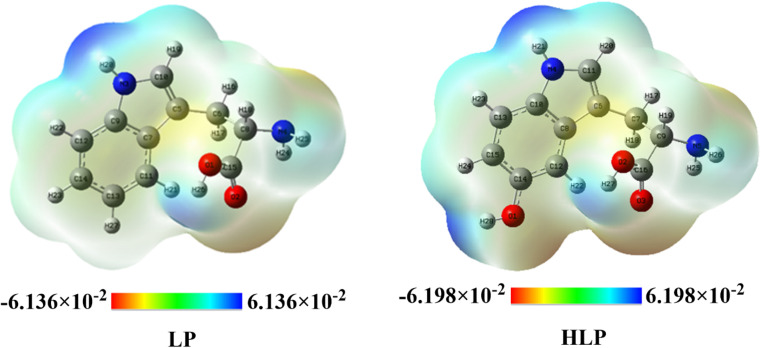
Molecular electrostatic potential of investigated derivatives.

To understand the reactivity of the studied derivatives, molecular electrostatic potential (MEP) analysis is conducted.^45,46^ The MEP surface visually represents the electron density distribution, with red regions indicating negatively charged areas prone to electrophilic attack,^47^ and blue regions denoting positively charged areas that attract radical molecules.^48^ Generally, in LP and HLP molecules, the oxygen in the carboxylic group exhibits high electrostatic potential, whereas the hydrogen atoms in the (OH) and (NH) groups display lower electrostatic potential (Fig. 7). Consequently, free radicals are more likely to attack the NH group in both LP and HLP molecules, as well as the OH group in HLP.

The blue color codes correspond to electrostatic potential values of 6.136 × 10^−2^ au for LP and 6.198 × 10^−2^ au for HLP, indicating that the hydrogen atoms in the OH and NH groups of HLP are the most susceptible to nucleophilic attacks.

#### 3.2.3. Thermodynamic parameters of LP and HLP in the gas phase and water

**Fig. 8:**
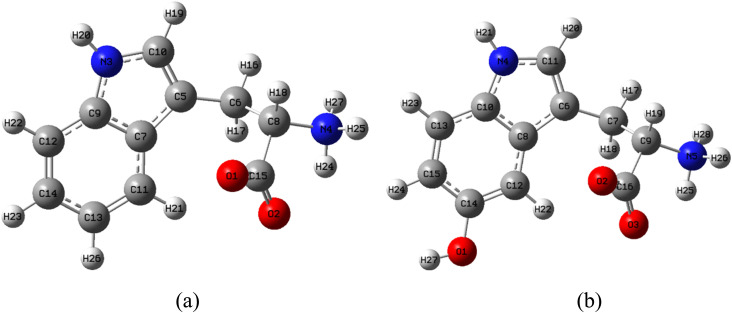
Zwitterionic forms of (a) LP and (b) HLP in water.

In the gas phase, LP and HLP exist as molecules, but their acidic groups can engage in acid–base equilibria in an aqueous medium. The components of corresponding species that exist in a medium depend on the investigated solution's pH value. Studies on the aqueous existence forms of LP and HLP have shown that they primarily exist as zwitterions, with mole fraction ratios of 0.990 for LP and 0.966 for HLP, respectively.^49^ Consequently, the zwitterionic forms of LP and HLP are selected for calculating thermodynamic parameters in an aqueous medium (Fig. 8).

To elucidate the mechanism of hydrogen atom transfer (HAT), the bond dissociation enthalpy (BDE) is calculated in Table 2. Given their lower BDE values, the amine and hydroxyl groups are identified as the most likely sites for hydrogen abstraction. In the gas phase, the N3-H20 bond in LP exhibits the lowest BDE of 91.5 kcal mol^−1^. For HLP, two bonds exhibit BDE values: O1-H27 at 83.4 kcal mol^−1^ and N4-H21 at 90.5 kcal mol^−1^. In an aqueous environment, the BDE values of these bonds generally increase, by 3.4 kcal mol^−1^ for LP and by 0.8 to 3.8 kcal mol^−1^ for HLP. This indicates that the ability of LP and HLP to donate hydrogen atoms decreases in water compared to the gas phase.

**Table 2:** Reaction enthalpies of LP and HLP at the M06-2X/6-311++G(d,p)

Thermodynamic parameters (kcal mol^−1^)	Gas			Water		
Bonds	N3-H20 (LP)	O1-H27 (HLP)	N4-H21 (HLP)	N3-H20 (LP)	O1-H27 (HLP)	N4-H21 (HLP)
BDE	91.5	83.4	90.5	94.9	84.2	94.3
IE	175.7	171.3		114.7	114.4	
PDE	229.3	225.4	232.6	17.7	7.3	17.4
PA	343.8	348.5	343.4	45.3	44.3	45.5
ETE	61.2	48.3	60.5	87.0	77.5	86.4

In the context of the single electron transfer-proton transfer (SELPT) mechanism, ionization energy (IE) and proton dissociation energy (PDE) are critical parameters. The IE values for LP and HLP are 175.7 and 171.3 kcal mol^−1^, respectively, in the gas phase (Table 2). These values decrease significantly to 114.7 kcal mol^−1^ for LP and 114.4 kcal mol^−1^ for HLP in water, demonstrating that the electron-donating ability of these compounds increases in an aqueous medium. The PDE value influences the proton dissociation of antioxidant molecule cations. For the N3-H20 bond in LP, the PDE value is 229.3 kcal mol^−1^ in the gas phase, which decreases sharply to 211.6 kcal mol^−1^ in water. A similar trend is observed for HLP, where the PDE values for the O1-H27 and N4-H21 bonds are 225.4 and 232.6 kcal mol^−1^, respectively, in the gas phase, decreasing to 215.2 and 218.1 kcal mol^−1^ in water.

Sequential proton loss electron transfer (SPLET) mechanism relates the proton affinity (PA) and electron transfer enthalpy (ETE) quantities. The PA values for the N3-H20 bond in LP and the O1-H27 and N4-H21 bonds in HLP are quite high in the gas phase (343.8, 348.5, and 343.4 kcal mol^−1^, respectively). These values decrease sharply in water (45.3, 44.3, and 45.5 kcal mol^−1^, respectively). In contrast, the ETE values increase in an aqueous medium for the studied bonds in LP and HLP, ranging from 25.8 to 29.2 kcal mol^−1^. This indicates that the electron donation process of the anion is more favorable in water than in the gaseous medium.

#### 3.2.4. Kinetics of reactions between LP, HLP and free radicals

**Fig. 9:**
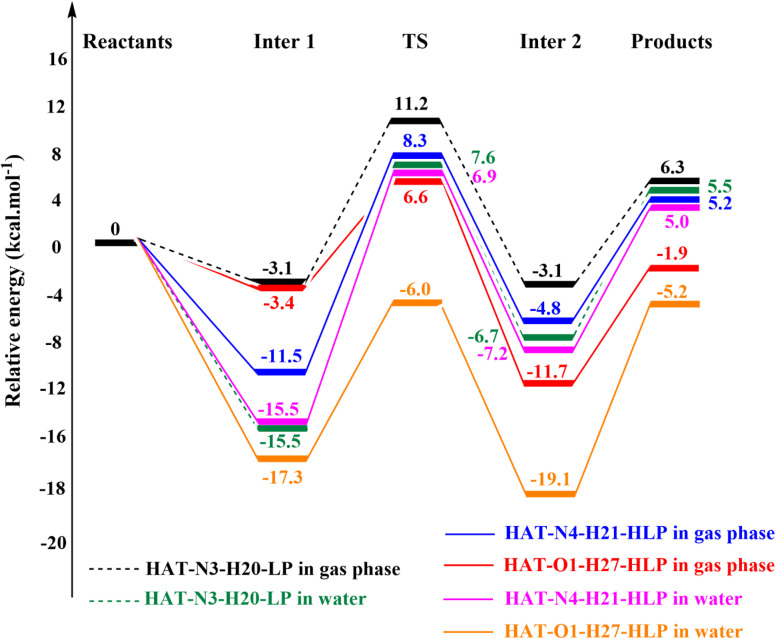
Potential energy surfaces for HOO˙ radical reactions with LP and HLP *via* HAT mechanism.

While thermochemical feasibility provides valuable insights into the viability of chemical processes, additional factors, such as reaction kinetics, are essential for evaluating the effectiveness of antioxidants. A promising antioxidant should be capable of reacting swiftly with free radicals. Therefore, the rate constants are computed for reactions between LP and HLP with the HOO˙ radical as the model substrate. These reactions proceed through a multistep mechanism involving the formation of intermediate species and transition states. As depicted in Fig. 9, the potential energy surfaces for these reactions of studied compounds are established in the gas phase and aqueous environments according to the HAT mechanism. The pathway involves the initial formation of intermediate 1 (Inter 1), followed by a transition state (TS) and, subsequently, intermediate 2 (Inter 2).^50^ The intermediates (Inter 1 and Inter 2) correspond to local minima on the potential energy surfaces, while the transition state (TS) serves as a saddle point linking these intermediates. To verify the connection between the TS and the intermediates, Intrinsic Reaction Coordinate (IRC) calculations were performed and shown in Fig. S1.† The reaction ultimately results in the formation of products, including hydrogen peroxide and a new stable radical.

For LP, the preferred reaction occurs at the N3-H20 bond. The energy values for Inter 1, TS, Inter 2, and products, relative to the reactants, are −3.1, 11.2, −3.1, and 6.3 kcal mol^−1^, respectively, in the gas phase. In water, these values decrease, corresponding to −15.5, 7.6, −6.7, and 5.5 kcal mol^−1^ due to the interaction between zwitterionic forms of LP and HOO˙.

In the case of HLP, the potential energy surface for the reaction at the O1-H27 bond shows values of −3.4, 6.6, −11.7, and −1.9 kcal mol^−1^ in the gas phase, and −17.3, −6.0, −19.1, and −5.2 kcal mol^−1^ in water, for Inter 1, TS, Inter 2, and products, respectively. For the N4-H21 bond in HLP, these energy values are generally higher than those for the O1-H27 bond. Specifically, the values for Inter 1, TS, Inter 2, and products are −11.5, 8.3, −4.8, and 5.2 kcal mol^−1^ in the gas phase, and −15.5, 6.9, −7.2, and 5.0 kcal mol^−1^ in water, respectively.

The analysis of singly occupied molecular orbitals (SOMO) at the transition states (TS) of hydrogen atom (H-atom) transfer from the O–H and N–H bonds in LP and HLP provides insights into the mechanism of the transfer process. It has been proposed that H-atom transfer (HAT) occurs when only one or no heteroatom is involved, whereas proton-coupled electron transfer (PCET) involves H-atom exchange between heteroatoms.^51^ To evaluate this distinction, the SOMO at the TSs of H-atom transfer from the N–H and O–H bonds in LP and HLP to the HOO˙ radical were examined, as depicted in Table S7 and Fig. S2 of ESI.† (ref. 52) However, further investigations are required to provide a more precise understanding of the mechanisms underlying HAT and PCET.

The kinetic parameters for the reactions between LP and HLP are summarized in Table 3. Employing conventional transition state theory, the rate constants for the hydrogen atom transfer (HAT) reactions are estimated using Eyringpy software. The results indicate that the reaction of LP with HOO˙ at the N3-H20 bond exhibits a rate constant of 7.83 × 10^2^ M^−1^ s^−1^ in the gas phase. For HLP, the rate constants are determined to be 1.14 × 10^5^ M^−1^ s^−1^ at the O1-H27 bond and 7.83 × 10^2^ M^−1^ s^−1^ at the N4-H21 position. Consequently, in the gas phase, HLP demonstrates a significantly higher total rate constant of 1.14 × 10^5^ M^−1^ s^−1^, suggesting that it reacts more rapidly with HOO˙ radicals compared to LP.

**Table 3:** Kinetic parameters for reactions of LP and HLP *via* HAT mechanism

Antioxidants	Bonds	Rate constants (M^−1^ s^−1^)			
		Gas		Water	
		*k*_HAT(i)_	*k*_HAT_	*k*_HAT(i)_	*k*_HAT_
LP	N3-H20	7.83 × 10^2^	7.83 × 10^2^	1.40 × 10^4^	1.40 × 10^4^
HLP	O1-H27	1.14 × 10^5^	1.14 × 10^5^	2.60 × 10^4^	3.70 × 10^9^
	N4-H21	7.83 × 10^2^		3.70 × 10^9^	

In water, the reactivity of both LP and HLP with free radicals increases significantly, with total rate constants of 1.40 × 10^4^ M^−1^ s^−1^ and 3.70 × 10^9^ M^−1^ s^−1^, respectively. Thus, HLP demonstrates a higher reactivity with HOO˙ compared to LP in both the gas and aqueous phases under the HAT mechanism.

The single electron transfer (SET) mechanism is employed to investigate the antioxidant ability of LP and HLP. The following reactions can represent the electron donation process of the studied antioxidants with HOO˙:

16 LP + HOO˙ → LP˙^+^ + HOO^−^

17 HLP + HOO˙ → HLP˙^+^ + HOO^−^

The reaction kinetics of LP and HLP with HOO˙ free radicals *via* the SET mechanism are summarized in Table 4. In the gas phase, the reaction enthalpies (Δ*H*°) are positive, with values of 152.87 kcal mol^−1^ for LP and 148.51 kcal mol^−1^ for HLP, indicating that these reactions are endothermic.^53,54^ It's noteworthy that the Gibbs free energy (Δ*G*°) for HLP (148.62 kcal mol^−^^1^) is lower than that for LP (152.26 kcal mol^−^^1^), suggesting that the electron donation reaction of HLP in the gas phase is thermodynamically more favorable than that of LP.

**Table 4:** Kinetic parameters for reactions of LP and HLP *via* SET mechanism

Phase	Compounds	Δ*G*^o^ (kcal mol^−1^)	Δ*H*^o^ (kcal mol^−1^)	*k*_SET_ (M^−1^ s^−1^)
Gas	LP	152.26	152.87	1.27 × 10^−276^
	HLP	148.62	148.51	8.13 × 10^−261^
Water	LP	37.29	37.72	1.48 × 10^−18^
	HLP	27.56	27.48	1.46 × 10^−16^

In water, both Δ*G*° and Δ*H*° values for LP and HLP are significantly reduced. Specifically, the Δ*G*° values are 37.29 kcal mol^−1^ for LP and 27.56 kcal mol^−1^ for HLP, while the Δ*H*° values are 37.72 kcal mol^−1^ for LP and 27.48 kcal mol^−1^ for HLP. This indicates that the electron donation reactions of these compounds are more favorable in water compared to the gas phase.

The electron exchange reaction (*k*_SET_) rate constants are determined using Marcus's theory.^55^ In the gas phase, the rate constant of LP in the reaction with the HOO˙ radical is 1.27 × 10^−276^ M^−1^ s^−1^, whereas that of HLP is 8.13 × 10^−261^ M^−1^ s^−1^*via* the SET mechanism. Interestingly, in water, the calculated rate constants for both LP and HLP increase to 1.48 × 10^−18^ M^−1^ s^−1^ and 1.46 × 10^−16^ M^−1^ s^−1^, respectively. These findings highlight the superior electron-donating ability of HLP compared to LP in gas and aqueous environments.

Using eqn (14) and (15), the product proportions (*P*%) formed through different reaction mechanisms are estimated and tabulated in Table 5. In both the gas phase and water, *P*_HAT_% values of LP and HLP are nearly 100%, indicating a strong predominance of HAT products over SET products.

**Table 5:** Product proportions formed through different reaction mechanisms in gas phase and water

Phase	Mechanism	LP	HLP
Gas	*P*_HAT_%	100.00	100.00
	*P*_SET_%	≈0.00	≈0.00
Water	*P*_HAT_%	100.00	100.00
	*P*_SET_%	≈0.00	≈0.00

## 4. Conclusions

The antioxidant ability of LP and HLP was evaluated *via* experimental methods and quantum chemical calculations. In the DPPH˙ assay, LP and HLP exhibited enhanced free radical scavenging abilities with increasing antioxidant concentrations. The IC_50DPPH_ of LP was 9.51 × 10^−3^ ± 0.53 × 10^−3^ M, whereas HLP demonstrated a significantly lower IC_50DPPH_ of 31.96 × 10^−7^ ± 0.85 × 10^−7^ M, indicating superior DPPH˙ free radical scavenging ability for HLP compared to LP. In the ABTS˙^+^ assay, LP had an IC_50ABTS_ value of 8.91 × 10^−4^ ± 0.73 × 10^−4^ M, while HLP exhibited an IC_50ABTS_ of 8.69 × 10^−6^ ± 0.95 × 10^−6^ M. This suggested that HLP more effectively neutralized ABTS˙^+^ free radical cations than LP. HOMO–LUMO analysis revealed that electron-donating positions were predominantly distributed across the aromatic rings and heteroatoms, while electron-accepting zones were localized at the nitrogen heteroatoms. Global reactivity descriptor values demonstrated the superior radical capturing reactivity of HLP compared to LP. Molecular electrostatic potential (MEP) investigations identified the hydrogen atoms in the hydroxyl and amine groups of the studied molecules as the most favorable positions for nucleophilic attacks. The combined thermodynamic and kinetic studies strongly suggested that the hydrogen atom transfer process was the primary mechanism by which LP and HLP scavenged free radicals. Although electron donation reactions of LP and HLP occurred more readily in water than in the gas phase, the SET mechanism was found to have a negligible impact on the overall antioxidant reactions of these compounds compared to the HAT mechanism. The calculated rate constants unequivocally demonstrated the superior free radical scavenging ability of HLP compared to LP. Theoretical analysis corroborated the notion that the presence of the hydroxyl group in the HLP molecule enhances its antioxidant properties.

## Data availability

The data supporting this article have been included as part of the ESI.†

## Author contributions

Dinh Quy Huong: conceptualization, methodology, validation, data curation, formal analysis, visualization, writing – original draft, writing – review & editing. Nguyen Quang Trung: data curation, formal analysis, visualization. Pham Dinh Tu Tai: data curation, formal analysis, visualization. Nguyen Minh Tam: methodology, validation, data curation. Pham Cam Nam: supervision, software, writing – review & editing. Nguyen Minh Thong: conceptualization, methodology, validation. Nguyen Hai Phong: supervision, writing – review & editing.

## Conflicts of interest

There are no conflicts of interest to declare.

## Supplementary Material

RA-014-D4RA06729K-s001
