# Supplementary material for: Investigation of the free radical scavenging ability of l-tryptophan and its derivatives using experimental methods and quantum chemical calculations
Source: RSC Adv. 2024 Nov 29;14(51):38059–69. doi: 10.1039/d4ra06729k (PMC11605380; doi:10.1039/d4ra06729k)
Supplement: RA-014-D4RA06729K-s001 [file RA-014-D4RA06729K-s001.pdf]

Supplementary Information (SI) for RSC Advances.
This journal is © The Royal Society of Chemistry 2024

Supplementary Data File

Investigation of free radical scavenging ability of L-Tryptophan and its derivatives using experimental methods and quantum chemical calculations

Dinh Quy Huong[1*], Pham Dinh Tu Tai[2], Nguyen Quang Trung[3,4], Nguyen Minh Thong[4], Nguyen Minh Tam[5], Nguyen Hai Phong[6], Pham Cam Nam[7]

[1]Department of Chemistry, University of Education, Hue University, Hue, Vietnam.

[2]Department of Planning, Finance and Facilities Management, Hue University, Hue, Vietnam.

[3]Quality Assurance and Testing Center 2, Danang, Vietnam.

[4]The University of Danang-University of Science and Education, Da Nang, Vietnam.

[5]Faculty of Basic Sciences, University of Phan Thiet, Phan Thiet, Vietnam.

[6]Department of Chemistry, University of Science, Hue University, Hue, Vietnam.

[7]Department of Chemical Engineering, The University of Danang – University of Science and Technology, Danang, Vietnam.

*: Corresponding author: dqhuong@hueuni.edu.vn

List of supporting information

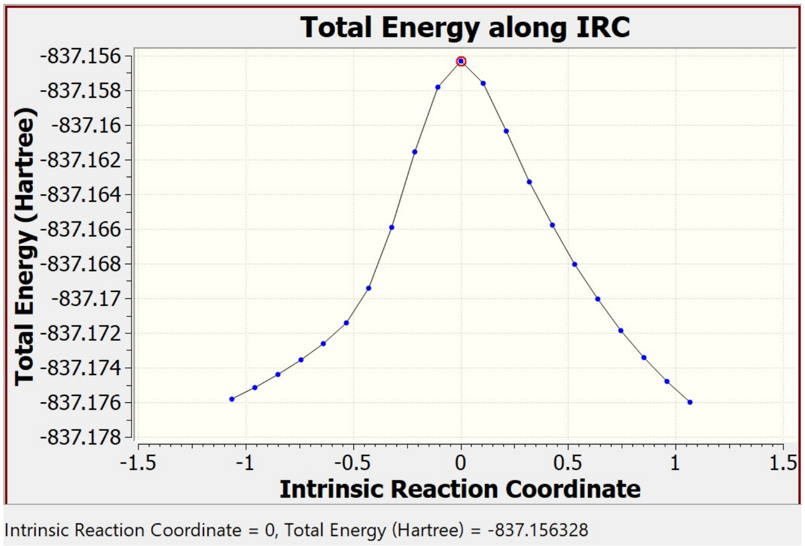

(a) N3-H20 (LP)

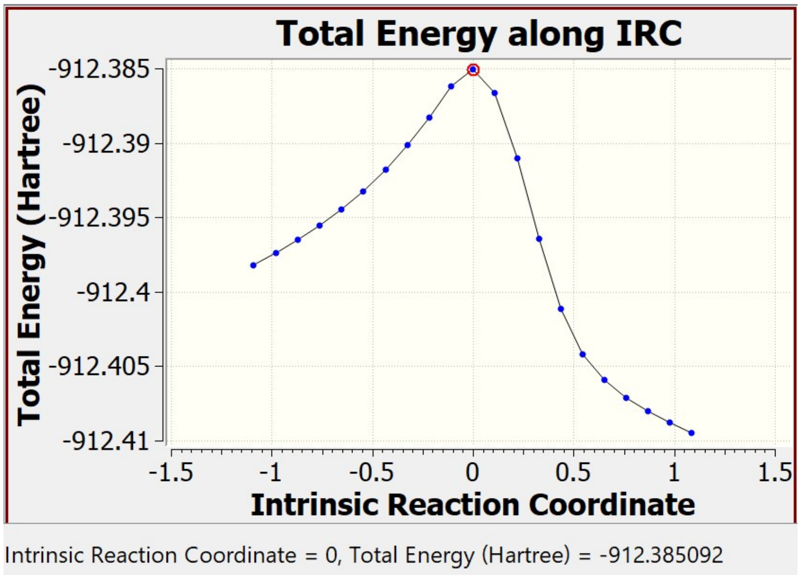

(b) O1-H27 (HLP)

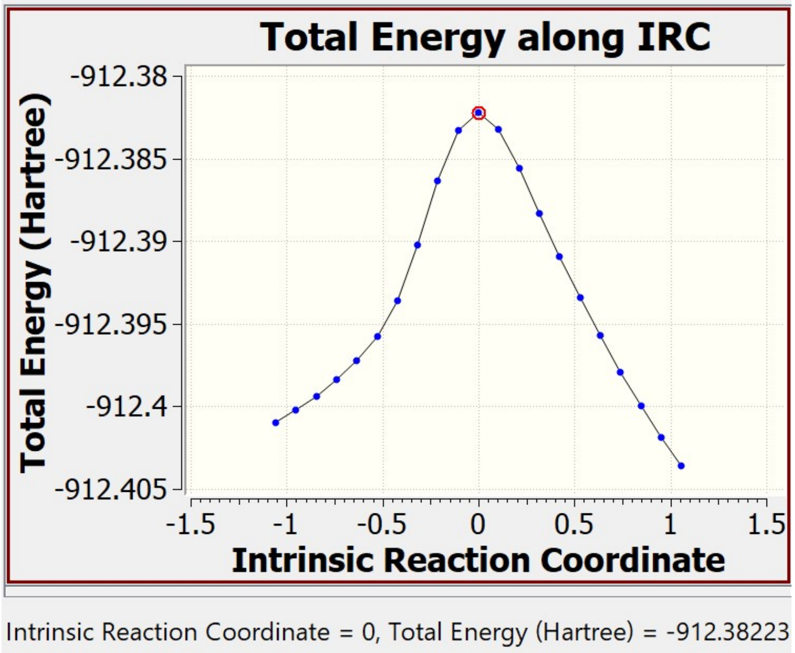

(c) N4-H21 (HLP)

Fig. S1. Intrinsic Reaction Coordinate of reactions between LP, HLP and HOO$^{\bullet}$ in the gas phase

Table S1. Detailed instructions on calculating the thermodynamic parameters for the HAT, SET-PT, SPLET mechanisms.

In the hydrogen atom transfer (HAT) mechanism, the transfer of hydrogen from the antioxidant molecule to the free radical is crucial, and this process is determined by the bond dissociation enthalpy (BDE) [20].

$$RXH + HOO^{\bullet} \rightarrow RX^{\bullet} + HOOH \qquad (1')$$

The BDE of RXH is calculated as follows:

$$BDE(RXH) = H(H^{\bullet}) + H(RX^{\bullet}) - H(RXH) \qquad (2')$$

In the case of the sequential electron transfer proton transfer (SET-PT) mechanism, two steps are involved [21]:

$$RXH + HOO^{\bullet} \rightarrow RXH^{\bullet+} + HOO^{-} \qquad (3')$$

$$RXH^{\bullet+} \rightarrow RX^{\bullet} + H^{+} \qquad (4')$$

These steps correspond to the thermodynamic parameters ionization potential (IP) and proton dissociation enthalpy (PDE), calculated as [22]:

$$IP = H(RXH^{\bullet+}) + H(e^{-}) - H(RXH) \qquad (5')$$

$$PDE = H(RX^{\bullet}) + H(H^{+}) - H(RXH^{\bullet+}) \qquad (6')$$

For the sequential proton-loss electron transfer (SPLET) mechanism [23]:

$$RXH \rightarrow RX^{-} + H^{+} \qquad (7')$$

$$RX^{-} + HOO^{\bullet} \rightarrow RX^{\bullet} + HOO^{-} \qquad (8')$$

Proton affinity (PA) and electron transfer enthalpy (ETE) are two critical parameters for SPLET [24], calculated as:

$$PA = H(RX^{-}) + H(H^{+}) - H(RXH) \qquad (9')$$

$$ETE = H(RX^{\bullet}) + H(e\text{-}) - H(RX^{-}) \qquad (10')$$

Where RXH represents LP and HLP, and X replaces O and N. The enthalpies of each species at 298 K were denoted by *H*. The enthalpies of the electron and proton in the gas phase and water were obtained from literature references [25].

Table S2. Optimized structures of LP forms in gas phase using M06-2X/6-311++g(d,p)

Stable Neutral form (1) - Gas				$C_{11}H_{12}N_2O_2$ - Gas
0 1				
O	1.23600200	-0.80056700	1.73337200	
O	2.13277900	-1.92852300	0.02257700	
N	-1.76837400	2.05075200	0.43691300	
N	3.80251600	0.36500800	-0.46293700	
C	0.09046900	1.16311400	-0.46650600	
C	1.49944400	0.99885300	-0.94468900	
C	-0.93687700	0.15264100	-0.42745300	
C	2.47443600	0.45408900	0.12073100	
C	-2.08405300	0.74432600	0.14718500	
C	-0.46337600	2.29287300	0.06507800	
C	-0.99085000	-1.19339800	-0.82352600	
C	-3.27280400	0.03646400	0.33909700	
C	-2.16507400	-1.89838200	-0.63319000	
C	-3.29539400	-1.28840400	-0.05618800	
C	1.95125500	-0.89148200	0.60441500	
H	1.90981400	1.95300300	-1.28114600	
H	1.52796200	0.31658900	-1.80190300	
H	2.49278700	1.14331800	0.96740400	
H	-0.02342700	3.26869700	0.20688000	
H	-2.39241600	2.72569600	0.84419100	
H	-0.12448600	-1.67520100	-1.26375700	
H	-4.14393400	0.50599900	0.78116700	
H	-4.20138900	-1.86716100	0.07811000	
H	3.81081300	-0.37614100	-1.15859900	
H	4.48725800	0.10982000	0.24049400	

H	0.84593300	-1.66915300	1.90746900
H	-2.22121900	-2.93721400	-0.93544800

**Stable Neutral form (2) - Gas**	**$C_{11}H_{12}N_2O_2$ - Gas**

0 1

O	1.23528100	-0.80156600	1.73240700
O	2.13408500	-1.92839500	0.02188000
N	-1.76930300	2.05065200	0.43675500
N	3.80233900	0.36589200	-0.46254100
C	0.09017900	1.16363000	-0.46594900
C	1.49930900	0.99986900	-0.94383800
C	-0.93682200	0.15279900	-0.42722000
C	2.47426800	0.45435800	0.12127200
C	-2.08441000	0.74408600	0.14700300
C	-0.46426000	2.29321800	0.06537500
C	-0.99009700	-1.19331400	-0.82312300
C	-3.27292800	0.03575500	0.33862600
C	-2.16408100	-1.89877600	-0.63302800
C	-3.29483600	-1.28918500	-0.05647800
C	1.95146600	-0.89167600	0.60397000
H	1.90962500	1.95434400	-1.27945800
H	1.52812000	0.31831600	-1.80161100
H	2.49249400	1.14294400	0.96847100
H	-0.02470800	3.26920300	0.20729500
H	-2.39369200	2.72536800	0.84388700
H	-0.12339400	-1.67484000	-1.26299900
H	-4.14439500	0.50497300	0.78037000
H	-4.20063000	-1.86830400	0.07761900
H	3.81069600	-0.37484200	-1.15863900

H	4.48705100	0.11025800	0.24075700
H	0.84560800	-1.67043500	1.90599100
H	-2.21968800	-2.93767900	-0.93513700

**Stable Neutral form (3) - Gas**	**$C_{11}H_{12}N_2O_2$ - Gas**

0 1

O	1.43830300	-0.91762500	1.46133200
O	3.56138900	-1.30669800	1.01550200
N	-1.58783600	1.94509100	0.61336700
N	3.70874100	0.31340000	-1.10320000
C	-0.01976900	0.77406300	-0.49797300
C	1.26575200	0.38133100	-1.17087100
C	-1.22922300	-0.01052300	-0.42755700
C	2.52286200	0.42847500	-0.27532400
C	-2.18623800	0.75383000	0.27571400
C	-0.29188100	1.95069700	0.15218900
C	-1.58166100	-1.28967200	-0.88790700
C	-3.47676800	0.28296300	0.53273500
C	-2.85576300	-1.75833500	-0.63717000
C	-3.79433800	-0.97802300	0.06643200
C	2.56574500	-0.68722100	0.77589500
H	1.46235300	1.04335100	-2.01837100
H	1.18115900	-0.62468600	-1.58924800
H	2.49101900	1.36306400	0.30924700
H	0.34140200	2.80932100	0.31799600
H	-2.01844300	2.68520300	1.14149300
H	-0.86600800	-1.89896200	-1.42793800
H	-4.19880200	0.88132500	1.07567700
H	-4.78527600	-1.37721400	0.24615100

H	4.47129600	-0.09215700	-0.57158500
H	3.99545100	1.21270000	-1.46737000
H	0.71304000	-0.35906900	1.14481700
H	-3.14338100	-2.74323100	-0.98368800

Stable Neutral form (4) - Gas	$C_{11}H_{12}N_2O_2$ - Gas

0 1

O	1.17226800	-0.89430100	1.63899500
O	2.48083700	-1.91465800	0.18229700
N	-1.79494600	2.02604300	0.44632000
N	3.83876800	0.36301200	-0.39681100
C	0.05850400	1.12004400	-0.45085500
C	1.45400700	0.94224300	-0.96550700
C	-0.98131600	0.12076100	-0.41993700
C	2.48197600	0.47848900	0.09970200
C	-2.12513400	0.72369400	0.14479800
C	-0.48560300	2.25650400	0.08539100
C	-1.03559700	-1.22776900	-0.80546700
C	-3.32281800	0.02671500	0.32565200
C	-2.21737600	-1.91987300	-0.63031700
C	-3.35048400	-1.29656100	-0.07178900
C	2.06330200	-0.88885700	0.63671000
H	1.82243200	1.88895800	-1.37193400
H	1.46512200	0.21478900	-1.78381700
H	2.45870100	1.19967300	0.92589100
H	-0.04207300	3.23207800	0.21952600
H	-2.41634300	2.70801200	0.84673600
H	-0.16051900	-1.72221800	-1.21428500
H	-4.19386100	0.50222700	0.76100000

H	-4.26192500	-1.86853800	0.05371400
H	4.04616300	1.09002900	-1.07101900
H	0.82656800	-0.00520400	1.78628800
H	3.96312900	-0.54141400	-0.84169100
H	-2.27574700	-2.96243400	-0.91760300

**Stable Neutral form (5) - Gas**	**$C_{11}H_{12}N_2O_2$ - Gas**

0 1

O	1.55331000	-0.57887300	1.98982000
O	1.89590700	-1.83662800	0.17007700
N	-1.64946300	2.05793500	0.44145000
N	3.80881100	0.04947500	-0.47969300
C	0.09954400	1.07619100	-0.57380200
C	1.47425300	0.80771200	-1.10410000
C	-0.97405500	0.12114000	-0.46825700
C	2.49139300	0.44539000	0.00444400
C	-2.05136500	0.77095200	0.17445500
C	-0.35839500	2.23197400	-0.00661300
C	-1.12610700	-1.21473800	-0.86974700
C	-3.26302600	0.12630900	0.43487600
C	-2.32321300	-1.85701100	-0.61460000
C	-3.38113700	-1.19174100	0.03521400
C	1.93614100	-0.67556300	0.86083300
H	1.86504400	1.68036800	-1.63609800
H	1.44139200	-0.01865500	-1.82108100
H	2.60440800	1.29693900	0.67778500
H	0.14055400	3.18133000	0.11764200
H	-2.20271900	2.75785000	0.90499600
H	-0.31464000	-1.73156700	-1.36995200

H	-4.07886600	0.63815200	0.93222000	
H	-4.30636500	-1.72284300	0.22377200	
H	4.22468500	0.79993800	-1.02007400	
H	3.72980000	-0.76206700	-1.08462600	
H	1.49994500	-2.50322100	0.74781800	
H	-2.45661100	-2.88777800	-0.92008800	
**Stable Neutral form (6) - Gas**				**$C_{11}H_{12}N_2O_2$ - Gas**
0 1				
O	1.27896300	-0.99333000	1.66259300	
O	2.44087400	-1.86794300	-0.02339800	
N	-1.89264200	2.02847900	0.40872500	
N	3.74243200	0.39848600	-0.43555200	
C	0.01311800	1.15655500	-0.39882100	
C	1.42501800	1.01995100	-0.87223000	
C	-0.99130600	0.12489900	-0.37008300	
C	2.42586300	0.53071000	0.19472600	
C	-2.17084100	0.70729200	0.14051000	
C	-0.58125600	2.28936200	0.08111900	
C	-0.99681100	-1.23262700	-0.72319200	
C	-3.35302200	-0.01942100	0.30036200	
C	-2.16352500	-1.95549100	-0.56862200	
C	-3.33103300	-1.35307600	-0.06209900	
C	1.99022500	-0.84137900	0.71571600	
H	1.78659300	1.98936700	-1.23170900	
H	1.46969200	0.33070800	-1.72332700	
H	2.40056100	1.21456400	1.04798600	
H	-0.16899100	3.27905600	0.20948900	
H	-2.54081800	2.69543000	0.79071900	

H	-0.09588300	-1.71364100	-1.08947400
H	-4.25055500	0.44140400	0.69620300
H	-4.22911500	-1.94858200	0.05046100
H	4.48863100	0.39627700	0.25139300
H	3.92035700	1.16066900	-1.07977700
H	3.09550700	-1.49784500	-0.64254600
H	-2.18178300	-3.00662000	-0.82949400

**Cation - Gas**	**$[C_{11}H_{12}N_2O_2]^{\bullet+}$ - Gas**

1 2

O	1.31713900	-0.47330100	1.89940400
O	1.84886500	-1.91461900	0.26744500
N	-1.60760000	2.08304100	0.41303700
N	3.76625000	0.10309300	-0.61432500
C	0.13159500	1.04982600	-0.58036000
C	1.50354200	0.83099300	-1.09289300
C	-0.92950300	0.11672300	-0.48673800
C	2.50590600	0.38744000	0.02060300
C	-2.02099700	0.76419600	0.13846100
C	-0.35928100	2.25951400	0.00322800
C	-1.05229500	-1.23058400	-0.87433000
C	-3.21582900	0.14141100	0.38996300
C	-2.25822800	-1.87799500	-0.62384000
C	-3.31481100	-1.20787800	-0.00902700
C	1.88218400	-0.80976800	0.73227400
H	1.91251300	1.73866300	-1.53970100
H	1.49675300	0.04516500	-1.85253700
H	2.62985500	1.19861400	0.74021000
H	0.15201700	3.20554300	0.11740800

H	-2.17960600	2.79341500	0.85314400	
H	-0.22546100	-1.75017200	-1.34486600	
H	-4.04747300	0.64364300	0.86882700	
H	-4.24459500	-1.73394300	0.17093200	
H	3.74377900	-0.79974300	-1.07950700	
H	4.53223600	0.10103100	0.04869300	
H	0.93833400	-1.27108200	2.29910100	
H	-2.38065700	-2.91397500	-0.91152700	
**Radical Form (N3-H20) - Gas**				**$[C_{11}H_{11}N_2O_2]^{\bullet}$ - Gas**
0 2				
O	1.21343600	-0.62962200	1.80175800	
O	1.98565000	-1.94225800	0.15991800	
N	-1.67027600	2.14687700	0.49627200	
N	3.77855600	0.21524900	-0.53470300	
C	0.10981600	1.10565500	-0.53087300	
C	1.49611300	0.90894600	-1.02773700	
C	-0.95202500	0.14770800	-0.47588200	
C	2.47475900	0.41420700	0.06828900	
C	-2.02357600	0.82004000	0.16415300	
C	-0.43867200	2.29059900	0.09075800	
C	-1.08332700	-1.17911900	-0.88286900	
C	-3.22309200	0.18446600	0.39989200	
C	-2.29983700	-1.82114400	-0.64241400	
C	-3.34886700	-1.15076800	-0.01387100	
C	1.88792500	-0.85770900	0.66857100	
H	1.91017400	1.84059700	-1.41902600	
H	1.50693000	0.16765400	-1.83374700	
H	2.54557200	1.17054700	0.85234800	

H	0.09295100	3.22723900	0.21903600	
H	-0.26576300	-1.70555200	-1.36400100	
H	-4.03652700	0.70482400	0.89090100	
H	-4.28287400	-1.67242400	0.15907400	
H	3.75252600	-0.60746100	-1.13123200	
H	4.48127000	0.04313400	0.17603000	
H	0.78130800	-1.45423900	2.06705700	
H	-2.43151800	-2.85146000	-0.94894600	
**Anion (N3-H20) - Gas**				**$[C_{11}H_{11}N_2O_2]^+$ - Gas**
-1 1				
O	1.15776200	-0.93297300	1.62532900	
O	2.19490400	-1.91750600	-0.09592200	
N	-1.83470200	2.13307600	0.47010500	
N	3.81500300	0.43108300	-0.34898800	
C	0.07015800	1.18581200	-0.44433000	
C	1.49592400	1.05552300	-0.87332500	
C	-0.94017300	0.18598300	-0.42403900	
C	2.45640800	0.45831500	0.18713200	
C	-2.08959600	0.83219600	0.15848700	
C	-0.54774000	2.31436300	0.10638700	
C	-1.01843700	-1.16808600	-0.80303600	
C	-3.27739900	0.10570500	0.34382200	
C	-2.20206600	-1.86257200	-0.60269900	
C	-3.32711600	-1.22754900	-0.02997200	
C	1.94433200	-0.92477000	0.54331600	
H	1.91472300	2.03286900	-1.13282000	
H	1.58090500	0.42104000	-1.76753300	
H	2.42232100	1.08703200	1.07894600	

H	-0.07306700	3.28345700	0.24517000	
H	-0.16308400	-1.67151400	-1.24773200	
H	-4.13933100	0.59788500	0.78447900	
H	-4.24145700	-1.79459700	0.11517500	
H	3.81839200	-0.18122000	-1.16092500	
H	4.44587200	0.00363200	0.32208900	
H	0.63553700	-1.74769500	1.60582700	
H	-2.27000000	-2.90568400	-0.89620800	
**Inter1-N3-H20-HAT- Gas**				**$C_{11}H_{12}N_2O_2$ + HOO$^{\bullet}$- Gas**
0 2				
O	-1.90210000	-0.17355600	1.84581000	
O	-3.55284900	0.23452900	0.39250900	
N	2.01334200	-0.02404300	-0.05108600	
N	-3.31328300	-2.50467700	-0.46254900	
C	-0.07214600	-0.54123900	-0.72192300	
C	-1.28167900	-1.32775100	-1.12051000	
C	0.01187200	0.88321300	-0.52400300	
C	-2.19808700	-1.72837100	0.05536200	
C	1.33147600	1.16716800	-0.10173900	
C	1.16522300	-1.04101800	-0.42501000	
C	-0.90212300	1.94005700	-0.66365100	
C	1.75800800	2.46689700	0.18475300	
C	-0.48347100	3.22677100	-0.37809200	
C	0.83516700	3.48691100	0.04332100	
C	-2.64460000	-0.45946400	0.76807200	
H	-0.99529400	-2.25639600	-1.61812300	
H	-1.88629000	-0.75425000	-1.83244600	
H	-1.62424300	-2.33675900	0.75706700	

H	1.51901200	-2.06050600	-0.45424700	
H	2.98319600	-0.14532400	0.20155000	
H	-1.92146200	1.75083700	-0.98242200	
H	2.77352000	2.66704400	0.50624500	
H	1.13231200	4.50652900	0.25800100	
H	-3.91190200	-1.89651600	-1.01517300	
H	-3.88507400	-2.86001000	0.29644800	
H	-2.16587500	0.70242900	2.16196800	
H	-1.17779900	4.05185200	-0.48231500	
O	4.76037700	-1.27586700	0.48604900	
O	4.22127400	-2.41328200	0.13896700	
H	4.92804300	-3.08152400	0.19273300	
**TS- N3-H20-HAT- Gas**				**$C_{11}H_{12}N_2O_2$ + HOO$^{\bullet}$- Gas**
0 2				
O	-2.08079500	0.34708600	1.73515900	
O	-3.37705800	0.90062200	-0.00399200	
N	1.84610000	-0.54680700	0.70952800	
N	-3.70257100	-1.92304700	-0.49731300	
C	-0.10854000	-0.74256000	-0.42667500	
C	-1.37801200	-1.33922100	-0.92946300	
C	0.25816200	0.65492200	-0.42448700	
C	-2.52038000	-1.34755300	0.11544200	
C	1.49099600	0.73184800	0.25738600	
C	0.91688700	-1.40645300	0.24361900	
C	-0.33281600	1.81238500	-0.92989300	
C	2.16798700	1.92690900	0.41792900	
C	0.34147600	3.02178800	-0.76505200	
C	1.57276600	3.07643700	-0.10823000	

C	-2.73001400	0.08331100	0.59463600
H	-1.22807500	-2.37617200	-1.23557400
H	-1.73308300	-0.78033200	-1.80219800
H	-2.21478500	-1.95840300	0.96697600
H	1.00189600	-2.46559600	0.44138500
H	2.97638900	-0.89199400	0.70014600
H	-1.29543500	1.77969800	-1.42856300
H	3.12189200	1.96685800	0.92998200
H	2.07566300	4.03007000	-0.00104800
H	-4.08987700	-1.26467400	-1.16797800
H	-4.42132100	-2.09487800	0.19723300
H	-2.17239400	1.29248100	1.92203100
H	-0.09508000	3.93393000	-1.15322900
O	4.08088300	-1.29744200	0.25591900
O	3.74987600	-2.49386100	-0.34479800
H	4.01519200	-2.36413900	-1.26424300

Frequencies -- -2555.3767 31.2234 39.8388

Red. masses -- 1.1404 6.9820 5.9234

Frc consts -- 4.3876 0.0040 0.0055

IR Inten -- 62171.0851 0.7277 0.2727

Atom	AN	X	Y	Z	X	Y	Z	X	Y	Z
1	8	0.00	0.00	0.00	0.15	-0.20	0.09	-0.10	0.17	-0.08
2	8	0.00	0.00	0.00	0.06	0.02	0.23	-0.14	-0.04	-0.11
3	7	-0.03	-0.02	0.00	-0.04	0.02	-0.13	0.09	-0.02	-0.17
4	7	0.00	0.00	0.00	-0.04	0.07	-0.05	0.02	-0.09	0.10
5	6	-0.02	0.02	-0.01	-0.03	0.05	-0.14	0.00	0.02	-0.02
6	6	0.00	0.00	0.00	-0.05	0.07	-0.13	-0.02	0.02	0.03
7	6	0.00	0.01	0.00	-0.03	0.05	-0.10	-0.01	0.03	0.03

8	6	0.00	0.00	0.00	0.01	-0.02	-0.06	0.00	0.03	0.05
9	6	-0.01	0.01	0.00	-0.03	0.03	-0.10	0.04	0.00	-0.06
10	6	0.04	0.01	0.02	-0.03	0.03	-0.15	0.06	0.00	-0.14
11	6	0.00	-0.01	0.00	-0.04	0.06	-0.06	-0.07	0.05	0.15
12	6	0.00	-0.01	0.00	-0.04	0.03	-0.07	0.04	0.00	-0.04
13	6	0.01	0.01	0.00	-0.05	0.06	-0.03	-0.07	0.05	0.18
14	6	-0.01	0.00	0.00	-0.04	0.04	-0.04	-0.02	0.02	0.08
15	6	0.00	0.00	0.00	0.07	-0.06	0.10	-0.09	0.05	-0.06
16	1	0.01	0.00	0.01	-0.06	0.10	-0.22	-0.02	0.01	0.04
17	1	0.00	0.00	0.00	-0.10	0.14	-0.06	-0.04	0.00	0.02
18	1	0.00	0.00	0.00	0.05	-0.11	-0.15	0.05	0.10	0.08
19	1	0.01	0.00	0.00	-0.03	0.03	-0.16	0.08	-0.01	-0.20
20	1	0.88	-0.34	-0.31	-0.04	0.02	-0.02	0.09	-0.01	-0.16
21	1	0.00	0.00	-0.01	-0.05	0.07	-0.05	-0.10	0.08	0.22
22	1	0.00	-0.01	0.00	-0.03	0.02	-0.07	0.08	-0.02	-0.11
23	1	0.00	0.00	0.00	-0.04	0.04	-0.02	-0.02	0.02	0.11
24	1	0.00	0.00	0.00	-0.06	0.15	0.04	-0.03	-0.16	0.06
25	1	0.00	-0.01	0.00	0.00	0.01	-0.03	0.04	-0.08	0.13
26	1	0.00	0.00	0.01	0.18	-0.22	0.20	-0.15	0.18	-0.15
27	1	0.00	0.00	0.00	-0.05	0.07	0.00	-0.11	0.07	0.27
28	8	-0.02	0.05	0.03	0.00	-0.02	0.11	0.10	-0.04	-0.10
29	8	-0.01	-0.03	-0.02	0.06	-0.14	0.32	0.14	-0.19	0.17
30	1	0.00	0.01	0.02	0.13	-0.32	0.32	0.17	-0.40	0.15

Zero-point correction= 0.232083 (Hartree/Particle)

Thermal correction to Energy= 0.248393

Thermal correction to Enthalpy= 0.249338

Thermal correction to Gibbs Free Energy= 0.185812

Sum of electronic and zero-point Energies= -836.924276

Sum of electronic and thermal Energies=	-836.907966		
Sum of electronic and thermal Enthalpies=	-836.907021		
Sum of electronic and thermal Free Energies=	-836.970547		
	E (Thermal)	CV	S
	KCal/Mol	Cal/Mol-Kelvin	Cal/Mol-Kelvin
Total	155.869	60.234	133.702
Electronic	0.000	0.000	1.377
Translational	0.889	2.981	42.291
Rotational	0.889	2.981	33.061
Vibrational	154.092	54.272	56.972
HF=-837.1563589			

**Inter2-N3-H20-HAT- Gas**	**$C_{11}H_{12}N_2O_2$ + HOO$^{\bullet}$- Gas**

0 2

O	-1.69547600	-0.29190900	1.87442300
O	-3.40899400	0.29826500	0.55870000
N	2.02921300	-0.08580900	-0.02893700
N	-3.33483400	-2.38831400	-0.50732500
C	-0.10642200	-0.45684200	-0.78470600
C	-1.31264300	-1.21878900	-1.19614000
C	0.01935600	0.95179800	-0.57522300
C	-2.17131900	-1.69298400	0.00573200
C	1.34242700	1.14568700	-0.10594500
C	1.18277900	-1.00049200	-0.41954800
C	-0.84984600	2.03348400	-0.71896400
C	1.81129500	2.39989700	0.21596700
C	-0.37501900	3.30419800	-0.39078700
C	0.93049900	3.48269000	0.06617400

C	-2.51941900	-0.46160900	0.83368100
H	-1.03963200	-2.11497500	-1.75667000
H	-1.95285100	-0.59950800	-1.83267300
H	-1.57826500	-2.37216900	0.62078200
H	1.47185400	-2.04479100	-0.45357200
H	3.61024900	-0.82116600	0.57558800
H	-1.86880500	1.89479100	-1.06389200
H	2.82358100	2.54067100	0.57469800
H	1.27356700	4.48061700	0.31216300
H	-3.98067900	-1.71606400	-0.91251800
H	-3.82918200	-2.86416800	0.23933000
H	-1.90715700	0.55484300	2.29353000
H	-1.02703900	4.16273200	-0.49279700
O	4.30812000	-1.48444200	0.76737800
O	3.89927400	-2.54129200	-0.09840800
H	4.61018200	-2.54914500	-0.74834400

**Anion (Adibatic) - Gas**	**$[C_{11}H_{12}N_2O_2]^-$ - Gas**

-1 2

O	1.17367800	-0.88181700	1.61967200
O	2.28228900	-1.98057400	0.02133700
N	-1.76872400	2.05236100	0.45030500
N	3.79338000	0.35191600	-0.44878600
C	0.08097600	1.15467900	-0.45722200
C	1.49038200	0.98796600	-0.92646100
C	-0.95206900	0.15477700	-0.42977600
C	2.46295900	0.43539100	0.14162100
C	-2.09595000	0.75560300	0.15361600
C	-0.46648800	2.28408000	0.08838400

C	-1.01673600	-1.18985000	-0.82718800	
C	-3.28794700	0.05307600	0.34399900	
C	-2.19616200	-1.88936100	-0.63548900	
C	-3.32188500	-1.27164700	-0.05582600	
C	1.99714400	-0.94314500	0.55986300	
H	1.89165800	1.95912800	-1.23601500	
H	1.54645600	0.32227300	-1.79306600	
H	2.40066500	1.08511300	1.02418900	
H	-0.02016600	3.25417400	0.25339800	
H	-2.36707400	2.73326800	0.90145300	
H	-0.15196300	-1.67491100	-1.26941200	
H	-4.15082600	0.53231800	0.79178000	
H	-4.23192600	-1.84505800	0.08005400	
H	4.44260500	-0.09723300	0.18883000	
H	4.13588500	1.29626700	-0.60530600	
H	0.77825200	-1.75693800	1.73034100	
H	-2.25924200	-2.92863600	-0.93808700	
**Anion (Vertical) - Gas**				**$[C_{11}H_{12}N_2O_2]^-$ - Gas**
-1 2				
O	1.23528100	-0.80156600	1.73240700	
O	2.13408500	-1.92839500	0.02188000	
N	-1.76930300	2.05065200	0.43675500	
N	3.80233900	0.36589200	-0.46254100	
C	0.09017900	1.16363000	-0.46594900	
C	1.49930900	0.99986900	-0.94383800	
C	-0.93682200	0.15279900	-0.42722000	
C	2.47426800	0.45435800	0.12127200	
C	-2.08441000	0.74408600	0.14700300	

C	-0.46426000	2.29321800	0.06537500	
C	-0.99009700	-1.19331400	-0.82312300	
C	-3.27292800	0.03575500	0.33862600	
C	-2.16408100	-1.89877600	-0.63302800	
C	-3.29483600	-1.28918500	-0.05647800	
C	1.95146600	-0.89167600	0.60397000	
H	1.90962500	1.95434400	-1.27945800	
H	1.52812000	0.31831600	-1.80161100	
H	2.49249400	1.14294400	0.96847100	
H	-0.02470800	3.26920300	0.20729500	
H	-2.39369200	2.72536800	0.84388700	
H	-0.12339400	-1.67484000	-1.26299900	
H	-4.14439500	0.50497300	0.78037000	
H	-4.20063000	-1.86830400	0.07761900	
H	3.81069600	-0.37484200	-1.15863900	
H	4.48705100	0.11025800	0.24075700	
H	0.84560800	-1.67043500	1.90599100	
H	-2.21968800	-2.93767900	-0.93513700	
**Cation (Vertical) - Gas**				**$[C_{11}H_{12}N_2O_2]^{+\bullet}$ - Gas**
1 2				
O	1.23528100	-0.80156600	1.73240700	
O	2.13408500	-1.92839500	0.02188000	
N	-1.76930300	2.05065200	0.43675500	
N	3.80233900	0.36589200	-0.46254100	
C	0.09017900	1.16363000	-0.46594900	
C	1.49930900	0.99986900	-0.94383800	
C	-0.93682200	0.15279900	-0.42722000	
C	2.47426800	0.45435800	0.12127200	

C	-2.08441000	0.74408600	0.14700300
C	-0.46426000	2.29321800	0.06537500
C	-0.99009700	-1.19331400	-0.82312300
C	-3.27292800	0.03575500	0.33862600
C	-2.16408100	-1.89877600	-0.63302800
C	-3.29483600	-1.28918500	-0.05647800
C	1.95146600	-0.89167600	0.60397000
H	1.90962500	1.95434400	-1.27945800
H	1.52812000	0.31831600	-1.80161100
H	2.49249400	1.14294400	0.96847100
H	-0.02470800	3.26920300	0.20729500
H	-2.39369200	2.72536800	0.84388700
H	-0.12339400	-1.67484000	-1.26299900
H	-4.14439500	0.50497300	0.78037000
H	-4.20063000	-1.86830400	0.07761900
H	3.81069600	-0.37484200	-1.15863900
H	4.48705100	0.11025800	0.24075700
H	0.84560800	-1.67043500	1.90599100
H	-2.21968800	-2.93767900	-0.93513700

Table S3. Optimized structures of HLP forms in gas phase using M06-2X/6-311++g(d,p)

**Stable Neutral form (1) - Gas**			**$C_{11}H_{12}N_2O_3$ - Gas**
0 1			
O	-2.63928300	-2.63279200	-0.54906900
O	1.29231700	-0.60445800	1.79460000
O	1.95073800	-2.10419300	0.27253000
N	-1.17396900	2.58272400	0.30144200
N	3.91615400	-0.29476100	-0.57129200

C	0.46555800	1.29220700	-0.53675300	
C	1.79592800	0.81364200	-1.02457900	
C	-0.71878300	0.49108100	-0.37816200	
C	2.69892700	0.18717600	0.06496400	
C	-1.72278500	1.32652200	0.15047700	
C	0.13649100	2.55083600	-0.11378300	
C	-1.00441800	-0.86219600	-0.63072900	
C	-3.00428600	0.84779300	0.43340200	
C	-2.27134400	-1.33100300	-0.34341300	
C	-3.26652900	-0.48427500	0.18448500	
C	1.96597500	-0.98122600	0.69669100	
H	2.35758200	1.64115400	-1.46818100	
H	1.67357000	0.05802700	-1.80585400	
H	2.85845300	0.93038800	0.85580200	
H	0.74533900	3.44175500	-0.07957600	
H	-1.65670500	3.39491700	0.64462000	
H	-0.24588800	-1.52737900	-1.03351200	
H	-3.77349500	1.49502600	0.83824700	
H	-4.24166300	-0.90903100	0.38642500	
H	4.43862500	-0.91808400	0.03317500	
H	4.51616400	0.48298400	-0.81941600	
H	-1.89068400	-3.12537300	-0.89618600	
H	0.74481500	-1.35191400	2.07331000	
**Stable Neutral form (2) - Gas**				**$C_{11}H_{12}N_2O_3$ - Gas**
0 1				
O	-2.56738500	-2.63613100	-0.58284400	
O	1.33948500	-0.70835000	1.80306100	
O	1.99783700	-2.09065300	0.17223200	

N	-1.16245000	2.58766800	0.30229300
N	3.99146800	-0.14060800	-0.52493300
C	0.46829000	1.27814400	-0.52774700
C	1.80573600	0.81704400	-1.01695300
C	-0.72207800	0.48392800	-0.36329600
C	2.71246300	0.20646600	0.07278700
C	-1.72117300	1.33519400	0.15916300
C	0.14695500	2.54132300	-0.11432000
C	-1.02086800	-0.86430200	-0.61251100
C	-3.00676900	0.87543100	0.43734400
C	-2.29627200	-1.31689200	-0.33429200
C	-3.28227100	-0.45709800	0.18742700
C	1.99823600	-0.99690600	0.67221500
H	2.36513800	1.64779600	-1.45147700
H	1.67983200	0.06643200	-1.80560800
H	2.86627200	0.94783000	0.85930600
H	0.76363700	3.42724800	-0.08940300
H	-1.63889400	3.40698500	0.63705400
H	-0.28584800	-1.55562600	-1.00893300
H	-3.77089300	1.53172900	0.83682300
H	-4.27334000	-0.84862700	0.39367800
H	3.85872200	-0.92134500	-1.16254400
H	4.64425500	-0.45568600	0.18486100
H	0.82326600	-1.48926300	2.04957700
H	-3.48825400	-2.81981400	-0.38336000

**Stable Neutral form (3) - Gas**	**$C_{11}H_{12}N_2O_3$ - Gas**

0 1

O	-3.36190700	-2.47456100	-0.45600800

O	1.65364600	-0.59651200	1.63293000	
O	3.67202000	-1.37682300	1.21166700	
N	-1.01081000	2.41243900	0.31509800	
N	3.90508800	-0.26849700	-1.20853200	
C	0.31480600	0.83474200	-0.59197000	
C	1.49414600	0.13666100	-1.20963800	
C	-0.99090400	0.26761500	-0.35950500	
C	2.79940700	0.18581600	-0.38617400	
C	-1.79190200	1.28064500	0.21088200	
C	0.24627000	2.13726900	-0.16245400	
C	-1.53407500	-1.00648100	-0.58096700	
C	-3.11987900	1.05297200	0.57013900	
C	-2.84973700	-1.22862400	-0.22747800	
C	-3.63736900	-0.20808100	0.34344900	
C	2.75648400	-0.68192800	0.87771300	
H	1.72320300	0.57569700	-2.18429400	
H	1.25200700	-0.91141700	-1.40238200	
H	2.93123700	1.21547200	-0.01423300	
H	1.00573200	2.90476700	-0.16972100	
H	-1.30114500	3.28954300	0.71270000	
H	-0.95679200	-1.81402400	-1.01418000	
H	-3.73163500	1.83124000	1.01028000	
H	-4.66811600	-0.42031500	0.60833700	
H	4.63518200	-0.66670200	-0.62792400	
H	4.29079300	0.48586400	-1.76155500	
H	-4.26689700	-2.51987900	-0.13864300	
H	0.99292900	-0.01830700	1.22297400	
**Stable Neutral form (4) - Gas**				**$C_{11}H_{12}N_2O_3$ - Gas**

0 1			
O	-2.70844700	-2.63387400	-0.59853900
O	1.29582800	-0.61867800	1.75109400
O	2.43057200	-2.07827900	0.54251000
N	-1.14719100	2.53815700	0.32079300
N	4.02497800	-0.19008300	-0.55555000
C	0.42707900	1.19160500	-0.55824600
C	1.72830400	0.68444700	-1.10066200
C	-0.78450600	0.43125100	-0.38084100
C	2.74585500	0.23009500	-0.02061300
C	-1.74762700	1.30433700	0.16763100
C	0.15272900	2.46114200	-0.11912000
C	-1.11577300	-0.90780300	-0.62943200
C	-3.04034300	0.87538000	0.46633000
C	-2.39781200	-1.32885400	-0.33870100
C	-3.35316400	-0.44499900	0.20310900
C	2.15892300	-0.93623100	0.77393000
H	2.21724800	1.46877800	-1.68619600
H	1.55200200	-0.15735100	-1.77776100
H	2.89758500	1.07026200	0.66844200
H	0.79009800	3.33287600	-0.10510000
H	-1.59507000	3.36819700	0.66950600
H	-0.39739400	-1.61852300	-1.02142500
H	-3.77976700	1.54570700	0.88787900
H	-4.35131000	-0.81243400	0.41874000
H	4.26882300	0.33614900	-1.38584800
H	-3.59444700	-2.82760300	-0.28372600
H	1.07071200	0.31951500	1.70729900

H	3.99140100	-1.17767200	-0.78933700	
**Stable Neutral form (5) - Gas**				**$C_{11}H_{12}N_2O_3$ - Gas**
0 1				
O	-2.73595400	-2.57057200	-0.55475100	
O	1.64401500	-0.53473200	2.01760700	
O	1.79940900	-1.96679100	0.30365900	
N	-1.04502500	2.57529800	0.28770600	
N	3.95346500	-0.45724200	-0.49705400	
C	0.47648000	1.19608100	-0.62941600	
C	1.76625600	0.63978800	-1.14761100	
C	-0.73975600	0.45901300	-0.40682100	
C	2.72995700	0.18144200	-0.02626100	
C	-1.66923700	1.35191600	0.17025600	
C	0.23962300	2.46974400	-0.18999500	
C	-1.11560700	-0.86925900	-0.65407800	
C	-2.95919700	0.94952100	0.51093100	
C	-2.39286300	-1.26593400	-0.31178300	
C	-3.30883500	-0.36652700	0.26731600	
C	2.01160300	-0.77568800	0.90485500	
H	2.29627900	1.38463500	-1.74885200	
H	1.56749100	-0.21693600	-1.79942900	
H	2.99575500	1.04293300	0.58869000	
H	0.89962100	3.32416200	-0.18035400	
H	-1.45437500	3.40671200	0.67715300	
H	-0.43533000	-1.58403200	-1.10108000	
H	-3.67000400	1.63626600	0.95493100	
H	-4.30405700	-0.71429900	0.52530700	
H	4.47090100	0.17544400	-1.09735700	

H	-3.64742900	-2.71854000	-0.29224900	
H	3.72951600	-1.28608200	-1.03910300	
H	1.30224200	-2.52048700	0.92129200	
**Stable Neutral form (6) - Gas**			**$C_{11}H_{12}N_2O_3$ - Gas**	
0 1				
O	-2.54768200	-2.69017500	-0.56476900	
O	1.36095600	-0.72117500	1.82539600	
O	2.20481600	-2.05966700	0.25872200	
N	-1.25641300	2.56685500	0.29606500	
N	3.90660700	-0.20066400	-0.55477600	
C	0.40468700	1.28044300	-0.49890300	
C	1.74028700	0.82893500	-0.99558400	
C	-0.77026000	0.46497200	-0.34098000	
C	2.69051400	0.29434600	0.09596000	
C	-1.79074200	1.30190800	0.15796800	
C	0.05832200	2.54219600	-0.10132600	
C	-1.03359000	-0.89111800	-0.57547000	
C	-3.07108700	0.81947400	0.42213100	
C	-2.30410800	-1.36563900	-0.31831000	
C	-3.31614100	-0.51892100	0.17447300	
C	2.02060100	-0.86459200	0.84003500	
H	2.25296000	1.66089800	-1.49005700	
H	1.61152400	0.04367900	-1.74932000	
H	2.86369500	1.08255400	0.83422400	
H	0.65676200	3.44057800	-0.07597800	
H	-1.74600700	3.37508100	0.63857900	
H	-0.27084500	-1.57822900	-0.92344400	
H	-3.85352000	1.46275900	0.80683300	

H	-4.30310800	-0.92807700	0.36545400
H	4.68380200	-0.25828900	0.09449500
H	-3.42445500	-2.92096700	-0.24956500
H	4.18774400	0.40403100	-1.31840400
H	2.87448600	-1.93123700	-0.43660300

Cation - Gas	$[C_{11}H_{12}N_2O_3]^{\bullet +}$ - Gas

1 2

O	-2.88854600	-2.50855000	-0.61057800
O	3.09530800	-1.15836200	1.66748800
O	1.36826900	-1.65707600	0.33669900
N	-1.19575000	2.46068000	0.49221600
N	3.95954000	0.05741500	-0.88765700
C	0.35942000	1.17272100	-0.51492300
C	1.63252800	0.71622600	-1.15737300
C	-0.87130400	0.41176400	-0.32321000
C	2.72050300	0.29380400	-0.16246900
C	-1.81045700	1.28398700	0.31139300
C	0.10865500	2.40840600	-0.00598700
C	-1.24956800	-0.86748600	-0.62657700
C	-3.12912200	0.88417200	0.64853600
C	-2.58132200	-1.26928300	-0.29216100
C	-3.50534200	-0.39289900	0.34196500
C	2.28749800	-0.93545100	0.62876200
H	2.05397700	1.52654400	-1.75452500
H	1.42656700	-0.11741000	-1.83393200
H	2.90216800	1.10253100	0.55105800
H	0.73899800	3.28319000	0.03880700
H	-1.61221400	3.27939200	0.91699400

H	-0.58140500	-1.59109200	-1.07606000
H	-3.81602800	1.56977400	1.13025200
H	-4.50160700	-0.74918000	0.57687800
H	3.88996700	-0.75480600	-1.49273400
H	4.73967400	-0.08424100	-0.25602200
H	2.83665000	-1.98740400	2.09490700
H	-3.79244700	-2.74783000	-0.36414000

**Radical Form (N4-H21) - Gas**	**$[C_{11}H_{11}N_2O_3]^{\bullet}$ - Gas**

0 2

O	-2.74007900	-2.51812300	-0.57943900
O	1.36218800	-0.56047100	1.84933800
O	1.87521400	-2.08428600	0.29109400
N	-1.04169800	2.66360000	0.33217500
N	3.95989600	-0.28737200	-0.58535700
C	0.49530200	1.21563100	-0.57938400
C	1.80197900	0.72342500	-1.08658300
C	-0.72460600	0.47121400	-0.39684500
C	2.72079800	0.15472800	0.02460600
C	-1.63679200	1.39698500	0.16467900
C	0.18703600	2.53662500	-0.10096000
C	-1.09063600	-0.84378300	-0.64572900
C	-2.92195800	1.01506800	0.47569600
C	-2.40257700	-1.22266800	-0.32459500
C	-3.30128100	-0.31239800	0.22487600
C	1.96102500	-0.96636200	0.72297200
H	2.36027200	1.52792600	-1.56979900
H	1.64703900	-0.07231300	-1.82321300
H	2.92902700	0.94213500	0.75155600

H	0.87824600	3.37175900	-0.09084100
H	-0.41216500	-1.57956500	-1.06369700
H	-3.62146000	1.72285700	0.90382200
H	-4.30948500	-0.63759500	0.46134100
H	3.78777600	-1.12979100	-1.12753500
H	4.64462500	-0.52637100	0.12338700
H	0.81586100	-1.28872500	2.17846500
H	-3.65546900	-2.67165900	-0.33156600

**Anion (N4-H21) - Gas**	**$[C_{11}H_{11}N_2O_3]^+$ - Gas**

-1 1			
O	-2.63995800	-2.59622600	-0.55386100
O	1.24387300	-0.85770400	1.69239700
O	2.07315200	-2.10166600	0.02760000
N	-1.20406100	2.68027100	0.33665700
N	4.03084100	-0.05781300	-0.40743500
C	0.46039000	1.30155900	-0.49612600
C	1.82345000	0.88235900	-0.94326000
C	-0.71026500	0.51536600	-0.35897400
C	2.70589900	0.21695000	0.14328400
C	-1.70327700	1.41893200	0.16385300
C	0.07585300	2.58050500	-0.05831100
C	-1.04225800	-0.83737700	-0.59860300
C	-2.99311400	0.94959200	0.43089400
C	-2.32431800	-1.26081100	-0.31758800
C	-3.30120400	-0.38391300	0.19614000
C	1.99580900	-1.04292600	0.60127600
H	2.39504400	1.74705000	-1.29495000
H	1.76464600	0.17256000	-1.78108100

H	2.79039400	0.90303300	0.98819100	
H	0.72654500	3.45221900	-0.03188200	
H	-0.32747100	-1.55000600	-1.00084400	
H	-3.74080400	1.63037300	0.82594900	
H	-4.29867200	-0.76320800	0.40691800	
H	3.92094800	-0.71870200	-1.17235700	
H	4.60484400	-0.52921000	0.28480800	
H	0.61511900	-1.59147600	1.74538400	
H	-3.57637800	-2.70648600	-0.37929100	
**Radical Form (O1-H27) - Gas**				**$[C_{11}H_{11}N_2O_3]^{\bullet}$ - Gas**
0 2				
O	-2.60141800	-2.64472200	-0.59524200	
O	1.33684400	-0.66717600	1.80495400	
O	2.03932400	-2.05708600	0.19884200	
N	-1.25538500	2.48926800	0.31483900	
N	3.97693100	-0.06975300	-0.53544200	
C	0.42408300	1.25591300	-0.53303300	
C	1.77109500	0.83205600	-1.02715700	
C	-0.74992000	0.42005100	-0.38060900	
C	2.69531200	0.25488700	0.06592500	
C	-1.77515400	1.24260400	0.15701500	
C	0.06799700	2.49715600	-0.10214400	
C	-1.01452700	-0.91361400	-0.64511900	
C	-3.07713800	0.76327800	0.44064600	
C	-2.32903400	-1.45008200	-0.37068700	
C	-3.33943100	-0.54648300	0.18292100	
C	2.00966800	-0.95759100	0.68221800	
H	2.30284600	1.67595200	-1.47036700	

H	1.66063500	0.07210700	-1.80855400
H	2.83677300	1.00904600	0.84267700
H	0.64967300	3.40506900	-0.05938000
H	-1.75592700	3.28796900	0.66834700
H	-0.27294600	-1.59338400	-1.05130100
H	-3.83302400	1.42525500	0.84845900
H	-4.31343400	-0.98060600	0.37321700
H	3.86578200	-0.87834900	-1.14157700
H	4.65089100	-0.33103900	0.17604600
H	0.86220900	-1.46579500	2.07835500
**Anion (O1-H27) - Gas**		**$[C_{11}H_{11}N_2O_3]^+$ - Gas**	
-1 1			
O	-2.54826300	-2.71698700	-0.55076500
O	1.13179600	-0.86865400	1.60205000
O	2.21248500	-2.09089300	0.06009200
N	-1.27090600	2.55775200	0.32867100
N	4.00778700	0.06664700	-0.35269200
C	0.40353000	1.30506700	-0.49438400
C	1.77928800	0.90394400	-0.92778300
C	-0.76541300	0.45696700	-0.36670800
C	2.66037700	0.26453900	0.17267600
C	-1.79018900	1.27735500	0.13872000
C	0.03929000	2.56242300	-0.08729900
C	-0.99672400	-0.90890100	-0.60900800
C	-3.06218900	0.75573500	0.41102600
C	-2.27479500	-1.49898000	-0.35521500
C	-3.28462600	-0.58077600	0.16942100
C	1.99973500	-1.03597700	0.59954200

H	2.33676100	1.77437400	-1.28553100
H	1.72057300	0.18934900	-1.75888700
H	2.69355100	0.94323000	1.02787900
H	0.62992600	3.46695200	-0.05460000
H	-1.82298200	3.38086400	0.48953200
H	-0.21988300	-1.54972000	-1.01770800
H	-3.85239700	1.38994800	0.80639000
H	-4.26029800	-1.01610700	0.36315400
H	3.95080000	-0.62259500	-1.09891300
H	4.59147300	-0.35894600	0.36105900
H	0.45644300	-1.56425200	1.53882600
**Inter1-N4-H21-HAT- Gas**		**$C_{11}H_{12}N_2O_3$ + HOO•- Gas**	
0 2			
O	-2.34757800	-2.24702100	-1.54652000
O	1.24276700	-1.10074900	1.32136600
O	2.20624700	-2.06427200	-0.45192700
N	-0.97649700	2.71350000	0.32796900
N	4.21676800	-0.02376500	-0.37557200
C	0.72766700	1.48659900	-0.48275500
C	2.10778100	1.07547400	-0.89464500
C	-0.49442800	0.73478000	-0.62931600
C	2.86447700	0.18290700	0.11377400
C	-1.53842400	1.53270000	-0.11017900
C	0.37831300	2.67597000	0.09871900
C	-0.78263000	-0.54770900	-1.12357400
C	-2.85799400	1.08268600	-0.06735300
C	-2.08872300	-0.99379200	-1.06865000
C	-3.11961300	-0.18448400	-0.54852900

C	2.09597000	-1.12006900	0.27995400
H	2.73541800	1.95564900	-1.04681900
H	2.06969000	0.53983700	-1.85028700
H	2.90108200	0.69364100	1.07840700
H	1.00440600	3.51654700	0.35793200
H	-1.47691200	3.48051100	0.74320700
H	-0.01682900	-1.19990400	-1.52859200
H	-3.65257500	1.69364200	0.34369700
H	-4.13292400	-0.57134300	-0.52076800
H	4.18435200	-0.60114700	-1.21179000
H	4.76951200	-0.53418800	0.30502600
H	0.67832400	-1.89457500	1.29382300
H	-3.25070200	-2.49470000	-1.33427800
O	-1.35132500	-1.61161200	1.98701000
O	-1.23449400	-0.33852600	2.24528100
H	-0.37406900	-0.06505200	1.85730900
**TS- N4-H21-HAT- Gas**		**$C_{11}H_{12}N_2O_3$ + HOO•- Gas**	
0 2			
O	2.84861900	2.88026300	0.24435100
O	-1.20597400	0.24940700	1.71745500
O	-1.72114900	2.28200600	0.94472700
N	0.92751300	-2.15934500	-0.93100000
N	-3.89007500	1.06696400	-0.51066500
C	-0.51289500	-0.41793600	-1.27353200
C	-1.79273200	0.30908700	-1.50235600
C	0.73663600	0.14024900	-0.79218300
C	-2.64749800	0.38151200	-0.21185400
C	1.58395800	-0.96139200	-0.56749800

C	-0.31874100	-1.78802300	-1.32966100
C	1.16295900	1.43574100	-0.52574400
C	2.85280900	-0.79494500	-0.05053100
C	2.45086700	1.60162600	-0.00990200
C	3.28052400	0.50443400	0.23122100
C	-1.83006600	1.09162500	0.86237000
H	-2.40606300	-0.19346000	-2.25189700
H	-1.60965200	1.33256900	-1.84571800
H	-2.86068300	-0.63708900	0.12058500
H	-1.04370300	-2.53720300	-1.61577000
H	0.81270500	-2.87925700	0.02778100
H	0.54208200	2.30804500	-0.69624300
H	3.49739400	-1.64488500	0.13781300
H	4.27341800	0.66679100	0.63826900
H	-3.70704600	2.05511500	-0.66137000
H	-4.53840200	0.99853600	0.26603500
H	-0.64350600	0.78606900	2.29628000
H	3.75037400	2.88314000	0.57547600
O	0.41140700	-3.11450100	1.19207100
O	-0.87237400	-2.64139800	1.21648500
H	-0.80815000	-1.74979700	1.59771500

Frequencies --	-2263.8431	35.1867	54.9330
Red. masses --	1.1221	6.7054	6.2430
Frc consts --	3.3881	0.0049	0.0111
IR Inten --	13593.4660	0.9610	0.1671

Atom	AN	X	Y	Z	X	Y	Z	X	Y	Z
1	8	0.00	0.00	0.00	-0.05	-0.09	0.19	-0.19	0.05	0.04
2	8	0.00	0.00	0.00	0.11	0.25	0.02	0.26	-0.01	-0.11

3	8	0.00	0.00	0.00	-0.10	0.14	-0.13	0.23	0.00	-0.06
4	7	-0.02	0.02	-0.03	0.01	-0.04	-0.12	0.00	-0.01	0.02
5	7	0.00	0.00	0.00	-0.06	-0.18	0.07	0.08	0.06	0.13
6	6	0.00	0.02	0.00	-0.01	-0.04	-0.02	-0.02	-0.05	-0.08
7	6	0.00	0.00	0.00	-0.03	-0.06	0.03	-0.04	-0.07	-0.04
8	6	0.00	0.01	0.00	-0.01	-0.05	0.00	-0.04	-0.01	-0.07
9	6	0.00	0.00	0.00	0.02	-0.04	0.06	0.07	0.01	0.04
10	6	0.00	0.00	0.00	0.00	-0.05	-0.07	-0.04	0.01	0.02
11	6	0.03	-0.03	0.01	0.01	-0.03	-0.09	0.00	-0.04	-0.04
12	6	-0.01	0.00	0.00	-0.03	-0.06	0.08	-0.08	0.00	-0.08
13	6	-0.01	0.00	0.00	0.00	-0.07	-0.06	-0.09	0.04	0.14
14	6	0.01	0.00	0.00	-0.03	-0.08	0.10	-0.14	0.04	0.03
15	6	0.00	0.01	0.00	-0.02	-0.08	0.03	-0.14	0.06	0.15
16	6	0.00	0.00	0.00	0.00	0.12	-0.03	0.19	0.00	-0.04
17	1	0.00	-0.01	0.00	-0.06	-0.10	0.07	-0.10	-0.11	0.05
18	1	0.01	0.00	0.01	-0.07	-0.07	-0.01	-0.07	-0.09	-0.12
19	1	0.00	0.00	0.01	0.13	-0.04	0.15	0.05	0.03	0.08
20	1	0.01	0.00	0.00	0.01	-0.03	-0.10	0.02	-0.07	-0.03
21	1	-0.31	-0.28	0.90	0.01	-0.04	-0.11	-0.02	-0.02	0.01
22	1	0.00	0.00	0.00	-0.03	-0.05	0.13	-0.08	-0.01	-0.17
23	1	-0.01	0.00	0.00	0.01	-0.07	-0.11	-0.10	0.06	0.23
24	1	0.00	0.00	0.00	-0.02	-0.10	0.05	-0.19	0.08	0.25
25	1	0.00	0.00	0.00	-0.16	-0.17	0.00	0.11	0.06	0.11
26	1	0.00	-0.01	0.00	-0.01	-0.19	0.10	0.14	0.10	0.17
27	1	-0.01	0.00	0.01	0.08	0.35	-0.05	0.37	-0.03	-0.19
28	1	0.00	0.00	0.00	-0.05	-0.10	0.19	-0.21	0.09	0.10
29	8	0.05	-0.01	-0.03	0.02	0.00	-0.09	-0.06	-0.05	-0.01
30	8	-0.03	0.01	0.00	0.12	0.27	0.00	-0.06	-0.05	-0.03

31 1 0.01 -0.03 0.00 0.32 0.25 0.01 -0.06 -0.05 -0.03

Zero-point correction= 0.236810 (Hartree/Particle)

Thermal correction to Energy= 0.253802

Thermal correction to Enthalpy= 0.254747

Thermal correction to Gibbs Free Energy= 0.191158

Sum of electronic and zero-point Energies= -912.145357

Sum of electronic and thermal Energies= -912.128364

Sum of electronic and thermal Enthalpies= -912.127420

Sum of electronic and thermal Free Energies= -912.191008

	E (Thermal)	CV	S
	KCal/Mol	Cal/Mol-Kelvin	Cal/Mol-Kelvin
Total	159.263	64.372	133.833
Electronic	0.000	0.000	1.377
Translational	0.889	2.981	42.486
Rotational	0.889	2.981	33.010
Vibrational	157.486	58.410	56.960

HF=-912.3821663

**Inter2-N4-H21-HAT- Gas**	**$C_{11}H_{12}N_2O_3$ + HOO•- Gas**

0 2

O	-2.96858800	-2.72657400	0.35701200
O	1.18930400	-0.57582600	1.72547200
O	1.71349800	-2.46726300	0.65872800
N	-0.95535500	2.24470200	-1.00372400
N	3.85428800	-0.97693100	-0.59368400
C	0.43773500	0.43865500	-1.30508800
C	1.71797400	-0.27598300	-1.53314500
C	-0.80078100	-0.07444600	-0.78969800
C	2.56887200	-0.40365800	-0.24143000

C	-1.62496200	1.06239400	-0.61116900	
C	0.22487300	1.86358100	-1.41062600	
C	-1.25179300	-1.35013100	-0.46708700	
C	-2.89822100	0.93671600	-0.10421700	
C	-2.54924500	-1.46808800	0.04523700	
C	-3.35710300	-0.34660000	0.22572300	
C	1.79714400	-1.27388100	0.74516100	
H	2.33806200	0.26145500	-2.25273500	
H	1.53989400	-1.28585900	-1.91801200	
H	2.71373500	0.59018500	0.18709800	
H	0.96840100	2.57659700	-1.74737200	
H	-0.37249500	3.17299400	0.70021700	
H	-0.64636100	-2.24135100	-0.59169000	
H	-3.52814000	1.80626600	0.03788100	
H	-4.35775100	-0.47217800	0.62680000	
H	3.74110200	-1.96068600	-0.82241700	
H	4.50225900	-0.91916700	0.18411200	
H	0.66772600	-1.19852000	2.25385300	
H	-3.87716000	-2.70266400	0.66868600	
O	0.08651000	3.16155400	1.55958000	
O	1.17213800	2.28195800	1.30125300	
H	0.88835600	1.45638700	1.71710400	
**Inter1-O1-H27-HAT- Gas**				**$C_{11}H_{12}N_2O_3$ + HOO$^{\bullet}$- Gas**
0 2				
O	-2.59698900	-1.13895300	-0.51722000	
O	1.76134800	-1.04505000	1.79393600	
O	1.78680100	-2.60674900	0.19225100	
N	0.91548400	2.99423600	0.27179900	

N	4.40582600	-1.68180500	-0.53047100	
C	1.82141900	1.10280900	-0.54245800	
C	2.82901400	0.10847100	-1.02876600	
C	0.40727900	0.89505000	-0.36088600	
C	3.39665100	-0.82097100	0.06548000	
C	-0.12653600	2.09773300	0.15103500	
C	2.07534900	2.38765200	-0.14895800	
C	-0.44131500	-0.20009900	-0.58553300	
C	-1.48274500	2.23335800	0.44261500	
C	-1.78655600	-0.06511700	-0.29495400	
C	-2.30394200	1.14346400	0.21697600	
C	2.24276900	-1.60425700	0.67539400	
H	3.68704200	0.61538100	-1.47479700	
H	2.38815100	-0.52415300	-1.80778300	
H	3.85313700	-0.20830300	0.84537100	
H	3.01173600	2.92520900	-0.14096000	
H	0.83757800	3.94306100	0.59377400	
H	-0.07575100	-1.14420600	-0.97371700	
H	-1.88863300	3.15866300	0.83472000	
H	-3.36464600	1.21308700	0.43323300	
H	3.95078400	-2.33492600	-1.16307000	
H	4.86143200	-2.24269700	0.18170600	
H	0.95642800	-1.52387500	2.03876200	
H	-3.50934200	-0.91114800	-0.30189600	
O	-5.42112600	-0.48119400	0.11559200	
O	-6.11736300	-1.54080800	-0.18467700	
H	-7.04677900	-1.31963900	0.00663600	

**TS-O1-H27-HAT- Gas**	**$C_{11}H_{12}N_2O_3$ + $HOO^{\bullet}$- Gas**

0 2			
O	-2.64752600	-1.23065300	0.18662500
O	1.94040400	-0.88940100	1.84027500
O	1.79921000	-2.52675300	0.32241700
N	0.73358500	3.00820400	0.09497100
N	4.31035500	-1.59521300	-0.73119200
C	1.63136900	1.08995300	-0.66668600
C	2.61667100	0.09272600	-1.18988800
C	0.25612900	0.85010900	-0.29078300
C	3.33643900	-0.72358100	-0.09561800
C	-0.26557200	2.08082700	0.18522200
C	1.86963600	2.40900400	-0.41785200
C	-0.56792400	-0.27118300	-0.32777500
C	-1.58582300	2.21249200	0.64372300
C	-1.88271300	-0.15989600	0.15528400
C	-2.37826100	1.09223300	0.63663500
C	2.28838300	-1.49420600	0.69619000
H	3.39787500	0.59173400	-1.76640900
H	2.11908200	-0.61413900	-1.86334200
H	3.84899900	-0.03500000	0.57911600
H	2.77084000	2.98274800	-0.57134700
H	0.65483500	3.97698500	0.35493700
H	-0.22929400	-1.23447600	-0.69310200
H	-1.96362300	3.16338100	1.00089000
H	-3.40273900	1.12952200	0.98695500
H	3.82183000	-2.32442700	-1.24426300
H	4.88104500	-2.06252200	-0.03497300
H	1.20831700	-1.39059100	2.22812500

H	-3.67763400	-0.96429700	-0.11189300
O	-4.73974400	-0.61469100	-0.69896800
O	-5.68608700	-1.52093800	-0.30604900
H	-5.97716000	-1.92123300	-1.13626100

Frequencies --	-2423.9109	27.6603	38.1921
Red. masses --	1.2024	6.5793	7.2412
Frc consts --	4.1621	0.0030	0.0062
IR Inten --	86632.7749	0.3312	0.8563

Atom	AN	X	Y	Z	X	Y	Z	X	Y	Z
1	8	-0.04	-0.03	-0.01	-0.05	-0.02	-0.03	-0.01	0.10	0.28
2	8	0.00	0.00	0.00	0.24	0.24	0.06	-0.03	-0.08	-0.02
3	8	0.00	0.00	0.00	-0.01	0.11	0.23	-0.07	-0.05	-0.05
4	7	0.01	0.01	0.00	0.01	-0.06	0.08	0.02	0.07	-0.08
5	7	0.00	0.00	0.00	-0.11	-0.11	-0.19	-0.08	-0.05	-0.07
6	6	-0.01	0.00	0.00	-0.03	-0.04	-0.02	-0.02	0.01	0.00
7	6	0.00	0.00	0.00	-0.07	-0.05	-0.08	-0.06	-0.02	-0.01
8	6	0.00	0.01	0.00	-0.02	-0.04	0.02	-0.01	0.05	0.07
9	6	0.00	0.00	0.00	0.03	-0.01	-0.12	-0.06	-0.05	-0.04
10	6	-0.01	-0.01	0.00	0.00	-0.06	0.08	0.02	0.08	0.01
11	6	0.00	0.01	0.00	-0.02	-0.05	0.02	0.00	0.03	-0.09
12	6	-0.02	0.00	0.01	-0.03	-0.04	0.00	-0.02	0.05	0.17
13	6	0.01	0.00	0.00	0.01	-0.06	0.11	0.03	0.12	0.04
14	6	0.04	0.05	-0.01	-0.03	-0.04	0.02	0.00	0.09	0.20
15	6	0.00	-0.02	-0.01	0.00	-0.05	0.07	0.02	0.13	0.12
16	6	0.00	0.00	0.00	0.08	0.12	0.08	-0.05	-0.06	-0.03
17	1	0.00	0.00	0.00	-0.13	-0.06	-0.17	-0.06	-0.04	-0.03
18	1	0.00	0.00	0.00	-0.14	-0.07	-0.01	-0.10	0.00	-0.01
19	1	0.00	0.00	0.00	0.16	0.01	-0.24	-0.04	-0.07	-0.03

20	1	0.00	0.00	0.00	-0.02	-0.05	0.01	0.00	0.01	-0.16
21	1	0.01	0.00	0.00	0.02	-0.07	0.11	0.04	0.08	-0.13
22	1	-0.01	0.00	0.00	-0.04	-0.03	-0.03	-0.04	0.03	0.21
23	1	0.00	0.00	0.00	0.03	-0.07	0.15	0.05	0.15	-0.01
24	1	0.01	0.01	0.02	0.00	-0.05	0.09	0.03	0.16	0.15
25	1	0.00	0.00	0.00	-0.23	-0.12	-0.06	-0.10	-0.03	-0.08
26	1	0.00	0.00	0.00	-0.04	-0.09	-0.23	-0.08	-0.08	-0.09
27	1	-0.01	0.01	-0.01	0.26	0.31	0.19	-0.03	-0.08	-0.01
28	1	0.88	-0.17	0.43	-0.03	0.00	-0.07	0.03	0.03	0.09
29	8	-0.06	-0.02	-0.01	0.01	0.03	-0.12	0.13	-0.13	-0.15
30	8	0.03	0.03	-0.01	-0.03	0.05	-0.17	0.13	-0.18	-0.24
31	1	-0.03	0.00	-0.02	0.01	0.05	-0.18	0.35	-0.31	-0.25

Zero-point correction= 0.236686 (Hartree/Particle)

Thermal correction to Energy= 0.254090

Thermal correction to Enthalpy= 0.255034

Thermal correction to Gibbs Free Energy= 0.188996

Sum of electronic and zero-point Energies= -912.148420

Sum of electronic and thermal Energies= -912.131016

Sum of electronic and thermal Enthalpies= -912.130072

Sum of electronic and thermal Free Energies= -912.196111

	E (Thermal)	CV	S
	KCal/Mol	Cal/Mol-Kelvin	Cal/Mol-Kelvin
Total	159.444	64.501	138.990
Electronic	0.000	0.000	1.377
Translational	0.889	2.981	42.486
Rotational	0.889	2.981	33.679
Vibrational	157.666	58.539	61.447

HF = -912.3851065

Inter2-O1-H27-HAT- Gas	$C_{11}H_{12}N_2O_3$ + HOO•- Gas

0 2

O	-2.52892000	-1.34420600	-0.84340300
O	1.76260100	-1.00850800	1.81683700
O	1.92472100	-2.47827600	0.13780600
N	0.52154800	2.92020000	0.35641900
N	4.50691900	-1.33927300	-0.44173800
C	1.68413200	1.19076500	-0.49310900
C	2.80856200	0.32259800	-0.96211700
C	0.28165600	0.83107300	-0.41325200
C	3.40881000	-0.58293700	0.13425700
C	-0.40325600	1.95299600	0.12965200
C	1.77824800	2.46246400	-0.01979300
C	-0.43374000	-0.30368700	-0.74738300
C	-1.80166800	1.96575800	0.35601600
C	-1.86206800	-0.32897700	-0.53772100
C	-2.50980200	0.85078600	0.03237000
C	2.30117200	-1.47464600	0.68083300
H	3.62932200	0.92918100	-1.34850400
H	2.46798300	-0.32142400	-1.78012800
H	3.78384300	0.04433000	0.94522200
H	2.64411500	3.09816600	0.08221900
H	0.32621100	3.83499700	0.72975300
H	0.02770600	-1.19257300	-1.16340000
H	-2.28599600	2.83894500	0.77813200
H	-3.57891500	0.78926700	0.19715600
H	4.13577100	-2.02876800	-1.09021200
H	5.00550700	-1.85316700	0.27673200

H	1.01854300	-1.58361100	2.04801100
H	-4.29483100	-1.28049600	-0.73480200
O	-5.24136100	-1.11927100	-0.53463700
O	-5.17820300	-0.80499900	0.85323100
H	-5.50150900	-1.61642400	1.25986400

**Anion (Adibatic) - Gas**	**$[C_{11}H_{12}N_2O_3]^-$ - Gas**

-1 2

O	-2.61403800	-2.62831600	-0.59570500
O	1.24255600	-0.78800500	1.67159500
O	2.18656000	-2.16653800	0.18711200
N	-1.16666300	2.58404300	0.32090600
N	3.98685900	-0.13416200	-0.50466900
C	0.45611100	1.27304400	-0.51638500
C	1.79588500	0.81510800	-0.99573000
C	-0.73492900	0.48530800	-0.36707700
C	2.70111400	0.19914500	0.09769700
C	-1.72984100	1.34058200	0.16794300
C	0.13770700	2.53475000	-0.08233200
C	-1.04520800	-0.86100300	-0.62464100
C	-3.01571500	0.88593000	0.44883900
C	-2.32181500	-1.30436600	-0.34166800
C	-3.30143400	-0.44407300	0.18916600
C	2.04601800	-1.05696900	0.62998300
H	2.34831900	1.66691400	-1.40657400
H	1.70394700	0.07511000	-1.79658800
H	2.76260100	0.91393500	0.92823300
H	0.75667000	3.41905800	-0.02986200
H	-1.61629700	3.39927200	0.72032700

H	-0.31523800	-1.55319900	-1.03128600	
H	-3.76768400	1.55003400	0.85811800	
H	-4.29384000	-0.83296800	0.39831200	
H	4.55251800	-0.68797900	0.12953800	
H	4.49294000	0.72629800	-0.69172400	
H	0.72316400	-1.58342400	1.85134100	
H	-3.53644500	-2.78408200	-0.38227800	
**Anion (Vertical) - Gas**				**$[C_{11}H_{12}N_2O_3]^-$ - Gas**
-1 2				
O	-2.56738500	-2.63613100	-0.58284400	
O	1.33948500	-0.70835000	1.80306100	
O	1.99783700	-2.09065300	0.17223200	
N	-1.16245000	2.58766800	0.30229300	
N	3.99146800	-0.14060800	-0.52493300	
C	0.46829000	1.27814400	-0.52774700	
C	1.80573600	0.81704400	-1.01695300	
C	-0.72207800	0.48392800	-0.36329600	
C	2.71246300	0.20646600	0.07278700	
C	-1.72117300	1.33519400	0.15916300	
C	0.14695500	2.54132300	-0.11432000	
C	-1.02086800	-0.86430200	-0.61251100	
C	-3.00676900	0.87543100	0.43734400	
C	-2.29627200	-1.31689200	-0.33429200	
C	-3.28227100	-0.45709800	0.18742700	
C	1.99823600	-0.99690600	0.67221500	
H	2.36513800	1.64779600	-1.45147700	
H	1.67983200	0.06643200	-1.80560800	
H	2.86627200	0.94783000	0.85930600	

H	0.76363700	3.42724800	-0.08940300	
H	-1.63889400	3.40698500	0.63705400	
H	-0.28584800	-1.55562600	-1.00893300	
H	-3.77089300	1.53172900	0.83682300	
H	-4.27334000	-0.84862700	0.39367800	
H	3.85872200	-0.92134500	-1.16254400	
H	4.64425500	-0.45568600	0.18486100	
H	0.82326600	-1.48926300	2.04957700	
H	-3.48825400	-2.81981400	-0.38336000	
**Cation (Vertical) - Gas**				**$[C_{11}H_{12}N_2O_3]^{+\bullet}$ - Gas**
1 2				
O	1.23528100	-0.80156600	1.73240700	
O	2.13408500	-1.92839500	0.02188000	
N	-1.76930300	2.05065200	0.43675500	
N	3.80233900	0.36589200	-0.46254100	
C	0.09017900	1.16363000	-0.46594900	
C	1.49930900	0.99986900	-0.94383800	
C	-0.93682200	0.15279900	-0.42722000	
C	2.47426800	0.45435800	0.12127200	
C	-2.08441000	0.74408600	0.14700300	
C	-0.46426000	2.29321800	0.06537500	
C	-0.99009700	-1.19331400	-0.82312300	
C	-3.27292800	0.03575500	0.33862600	
C	-2.16408100	-1.89877600	-0.63302800	
C	-3.29483600	-1.28918500	-0.05647800	
C	1.95146600	-0.89167600	0.60397000	
H	1.90962500	1.95434400	-1.27945800	
H	1.52812000	0.31831600	-1.80161100	

H	2.49249400	1.14294400	0.96847100
H	-0.02470800	3.26920300	0.20729500
H	-2.39369200	2.72536800	0.84388700
H	-0.12339400	-1.67484000	-1.26299900
H	-4.14439500	0.50497300	0.78037000
H	-4.20063000	-1.86830400	0.07761900
H	3.81069600	-0.37484200	-1.15863900
H	4.48705100	0.11025800	0.24075700
H	0.84560800	-1.67043500	1.90599100
H	-2.21968800	-2.93767900	-0.93513700

Table S4. Optimized structures of LP forms in water using M06-2X/6-311++g(d,p)

**Stable Neutral form - Water**			**$C_{11}H_{12}N_2O_2$ - Water**
0 1			
O	1.33267600	-0.89124100	1.78425000
O	2.39766200	-1.85467400	0.04804800
N	-1.87172300	2.02177800	0.42903900
N	3.73526000	0.30268900	-0.48735500
C	0.01289400	1.13579500	-0.41486900
C	1.41692900	0.98174700	-0.90951000
C	-1.00221000	0.11638200	-0.38315200
C	2.39793300	0.50180200	0.16896400
C	-2.17046400	0.70985500	0.14972700
C	-0.56428100	2.27184600	0.08378800
C	-1.02938400	-1.23649600	-0.75725900
C	-3.36094500	-0.00633900	0.31428200
C	-2.20504500	-1.94812900	-0.59784600
C	-3.36016600	-1.33690400	-0.06669100

C	1.99872700	-0.88861000	0.74166500
H	1.77403100	1.93759200	-1.30420700
H	1.43653100	0.25891900	-1.73306500
H	2.50785400	1.23955600	0.96109700
H	-0.14147300	3.25640100	0.21600800
H	-2.51175300	2.70266700	0.80632100
H	-0.13767900	-1.71532800	-1.14834400
H	-4.24737000	0.46366900	0.72382800
H	-4.26516300	-1.92209400	0.04667200
H	3.69610100	-0.64675200	-0.89324700
H	4.49844200	0.31581700	0.19101200
H	-2.24374100	-2.99361100	-0.88016000
H	3.94283500	0.99352600	-1.21068300
**Cation - Water**		**$[C_{11}H_{12}N_2O_2]^{\bullet+}$ - Water**	
1 2			
O	1.34165800	0.68183900	1.43308000
O	3.43493900	-0.12608200	1.64169000
N	-1.35914400	1.94268000	-0.39804400
N	3.68028800	-0.82743700	-0.81943600
C	0.00285800	0.19128700	-0.75555500
C	1.21627200	-0.58214700	-1.12412400
C	-1.25993200	-0.32010200	-0.34309500
C	2.48593700	0.02349800	-0.53116800
C	-2.10613700	0.78535000	-0.11364800
C	-0.13746400	1.61704500	-0.77463600
C	-1.74558700	-1.61688800	-0.13055000
C	-3.40724400	0.65980200	0.31557900
C	-3.05890700	-1.76215500	0.29865300

C	-3.87239200	-0.64590100	0.51785300
C	2.41016700	0.20272900	1.01801700
H	1.29535100	-0.61416700	-2.21767700
H	1.09631500	-1.61178700	-0.77749800
H	2.69304000	1.00298900	-0.96408400
H	0.59238000	2.36849300	-1.03562100
H	-1.70472200	2.89213700	-0.32107300
H	-1.11737600	-2.48352400	-0.29573200
H	-4.04019600	1.51973500	0.49092300
H	-4.89107500	-0.79074300	0.85529900
H	4.28522600	-0.72098800	0.01619100
H	4.18333100	-0.55843800	-1.66576800
H	-3.46024100	-2.75254100	0.46944700
H	3.44176200	-1.81902500	-0.89416600

**Radical Form (N3-H20) - Water**	**$[C_{11}H_{11}N_2O_2]^{\bullet}$ - Water**

0 2

O	1.29600000	-0.47065200	1.80955900
O	2.67413600	-1.78873000	0.60698200
N	-1.65002900	2.09162400	0.51582800
N	3.73699000	0.14270900	-0.70791700
C	0.01608300	0.90556500	-0.54124600
C	1.35301900	0.62143900	-1.12595600
C	-1.11543200	0.03993400	-0.46232300
C	2.43539600	0.47600000	-0.04310500
C	-2.11820900	0.79526500	0.19631300
C	-0.41827600	2.13684600	0.08365900
C	-1.35867200	-1.27452500	-0.86007800
C	-3.36090400	0.25682900	0.45721200

C	-2.61667400	-1.81840200	-0.59551300
C	-3.59901500	-1.06572900	0.05032500
C	2.10394000	-0.71488500	0.90560100
H	1.63389000	1.44287900	-1.79423400
H	1.31527600	-0.29792000	-1.71745600
H	2.55592600	1.39982900	0.51877800
H	0.19145800	3.02662400	0.19300000
H	-0.59661000	-1.86297900	-1.35932100
H	-4.12618600	0.83448500	0.96191600
H	-4.56717600	-1.51214400	0.24359600
H	3.77283900	-0.89373200	-0.70023900
H	4.53546500	0.48255100	-0.16952000
H	-2.83471100	-2.83651400	-0.89326200
H	3.82247600	0.50162900	-1.66029600
**Anion (N3-H20) - Water**		**$[C_{11}H_{11}N_2O_2]^+$ - Water**	
-1 1			
O	1.47520400	-0.98356000	1.79884000
O	2.45774300	-1.84511000	-0.03460200
N	-1.91557200	2.09422300	0.50625900
N	3.72684600	0.37951700	-0.50695300
C	-0.02357100	1.11913400	-0.39731800
C	1.37728400	0.96479400	-0.89979500
C	-1.05084000	0.13477900	-0.37591900
C	2.38422500	0.50365400	0.16416900
C	-2.19423200	0.79390200	0.19178200
C	-0.61696300	2.26025600	0.14311000
C	-1.13833700	-1.21511600	-0.76687200
C	-3.40131600	0.08636400	0.35244900

C	-2.33682800	-1.88911600	-0.60113500
C	-3.46363200	-1.23980500	-0.04273800
C	2.06583800	-0.91529300	0.71222100
H	1.73386800	1.91898400	-1.30486800
H	1.40585500	0.23537200	-1.71903600
H	2.47194700	1.22995100	0.96992700
H	-0.13078200	3.22297500	0.27515900
H	-0.27149400	-1.72492100	-1.17931200
H	-4.26853900	0.57956000	0.78219100
H	-4.38854600	-1.79376800	0.07772500
H	3.71394600	-0.54227800	-0.97025700
H	4.49507400	0.37638500	0.16553400
H	-2.41706900	-2.92968000	-0.89696900
H	3.90347700	1.11929500	-1.18886700

**Inter1-N3-H20-HAT - Water**	**$C_{11}H_{12}N_2O_2$ + HOO$^{\bullet}$- Water**

0 2

O	-1.24151300	-0.51719100	-1.64568300
O	-2.63688900	-1.84060800	-0.50237600
N	1.80506100	1.79121400	0.80482700
N	-3.87188000	0.14940800	0.62970400
C	-0.13009400	0.70570500	1.14545500
C	-1.56908700	0.43308400	1.45437500
C	0.88114600	-0.25605300	0.79779700
C	-2.45678500	0.40715300	0.20107400
C	2.08134000	0.46280400	0.59085300
C	0.48114200	1.93117100	1.13520400
C	0.88198300	-1.65109000	0.63584700
C	3.27825600	-0.16941600	0.23858300

C	2.06222700	-2.28035800	0.28398000	
C	3.25022400	-1.54428600	0.08828700	
C	-2.08093700	-0.76097500	-0.74417000	
H	-1.94931300	1.20630500	2.12797100	
H	-1.66922800	-0.52887400	1.96906000	
H	-2.42928600	1.36549100	-0.31537500	
H	0.06676800	2.90603000	1.34407300	
H	2.46272400	2.54941400	0.71411400	
H	-0.03138200	-2.22194800	0.77044000	
H	4.18843400	0.39829400	0.08452500	
H	4.15828600	-2.06746300	-0.18754700	
H	-3.96045600	-0.86974700	0.75492500	
H	-4.54363200	0.42610300	-0.08912400	
O	1.29812900	1.02697500	-2.36977400	
O	0.15510100	1.52964100	-1.98725300	
H	-0.45979900	0.71783400	-1.78522000	
H	-4.12038200	0.63306900	1.49494600	
H	2.07989900	-3.35583100	0.15248400	
**TS-N3-H20-HAT- Water**				**$C_{11}H_{12}N_2O_2$ + HOO•- Water**
0 2				
O	-1.08551700	0.31730400	-1.65632000	
O	-2.31264200	-1.53777400	-1.36574500	
N	1.64908300	1.46956100	1.00250000	
N	-3.80256700	-0.47376700	0.48221600	
C	-0.15020300	0.08684200	1.27626500	
C	-1.55977500	-0.34345700	1.49498700	
C	0.90376500	-0.69969500	0.68438100	
C	-2.43602300	0.11854200	0.31398900	

C	1.98668200	0.18338200	0.50761500
C	0.36788000	1.36439300	1.44865900
C	0.99982000	-2.02742300	0.27086600
C	3.16337200	-0.22054200	-0.09412100
C	2.18732200	-2.44320900	-0.32921300
C	3.24944000	-1.55318000	-0.51158700
C	-1.89471800	-0.41479600	-1.04392100
H	-1.95482400	0.08806900	2.41831900
H	-1.61718200	-1.43300100	1.57324900
H	-2.52947600	1.20313500	0.30446600
H	-0.14277400	2.22223300	1.86237400
H	1.63364000	2.23032400	0.06706100
H	0.17632200	-2.71977000	0.40718500
H	3.98644600	0.46909100	-0.23920900
H	4.15982000	-1.90210300	-0.98410800
H	-3.75544900	-1.42341800	0.08145700
H	-4.50753600	0.04488900	-0.04546200
O	1.19066700	2.62146700	-1.04929900
O	-0.16346300	2.62329000	-0.87047600
H	-0.49410800	1.73529200	-1.20779700
H	-4.10856100	-0.51524000	1.45631800
H	2.29033600	-3.46950400	-0.65966300

Frequencies -- -2780.1092 48.6184 56.7087

Red. masses -- 1.1799 6.5930 6.5025

Frc consts -- 5.3731 0.0092 0.0123

IR Inten -- 66495.3737 14.3019 24.0742

Atom	AN	X	Y	Z	X	Y	Z	X	Y	Z
1	8	0.00	0.01	-0.00	0.14	-0.08	0.00	-0.06	0.25	0.06

2	8	0.00	0.00	-0.00	0.29	-0.16	0.14	0.23	0.04	-0.03
3	7	0.03	0.01	-0.03	0.03	0.01	0.05	-0.03	-0.02	-0.13
4	7	-0.00	0.00	-0.00	0.05	-0.01	-0.11	0.03	-0.22	-0.01
5	6	0.01	0.03	0.01	-0.02	0.08	0.08	-0.01	-0.01	0.00
6	6	-0.00	0.00	0.00	-0.04	0.12	0.06	-0.00	-0.03	0.04
7	6	0.00	0.00	0.00	-0.07	0.05	0.02	-0.03	-0.04	0.02
8	6	0.00	-0.00	0.00	0.04	0.01	-0.05	-0.03	-0.06	0.05
9	6	-0.00	0.01	-0.00	-0.05	0.01	-0.00	-0.04	-0.04	-0.07
10	6	-0.05	-0.03	0.01	0.04	0.05	0.09	-0.02	0.00	-0.09
11	6	0.00	-0.00	-0.00	-0.16	0.06	-0.04	-0.02	-0.08	0.13
12	6	0.01	-0.01	-0.01	-0.10	-0.02	-0.09	-0.05	-0.08	-0.08
13	6	-0.01	-0.00	0.00	-0.21	0.04	-0.13	-0.04	-0.12	0.14
14	6	0.00	0.01	0.00	-0.19	-0.00	-0.16	-0.05	-0.12	0.03
15	6	-0.00	-0.00	0.01	0.17	-0.09	0.04	0.06	0.09	0.03
16	1	-0.01	-0.01	-0.00	-0.08	0.23	-0.01	-0.00	-0.04	0.04
17	1	-0.01	-0.00	0.00	-0.06	0.13	0.17	0.03	-0.03	0.04
18	1	-0.00	-0.00	0.00	0.02	0.00	-0.14	-0.16	-0.07	0.11
19	1	-0.01	0.00	-0.01	0.08	0.07	0.11	-0.00	0.02	-0.11
20	1	0.29	-0.40	0.85	0.01	0.02	0.05	0.00	-0.00	-0.10
21	1	-0.00	0.00	0.00	-0.19	0.10	-0.03	-0.01	-0.07	0.22
22	1	0.00	-0.00	-0.00	-0.08	-0.05	-0.12	-0.06	-0.09	-0.15
23	1	0.00	0.00	-0.00	-0.23	-0.02	-0.23	-0.06	-0.15	0.03
24	1	-0.00	0.00	0.00	0.11	-0.05	-0.01	0.15	-0.20	-0.05
25	1	-0.00	-0.00	0.00	0.08	-0.08	-0.23	-0.02	-0.28	0.01
26	8	-0.06	0.01	-0.02	-0.00	0.03	0.06	0.01	0.09	-0.07
27	8	0.04	0.00	-0.01	-0.00	-0.05	0.05	0.01	0.26	-0.02
28	1	-0.05	-0.11	-0.05	0.05	-0.06	0.02	-0.08	0.29	0.03
29	1	0.01	0.00	0.00	-0.04	0.08	-0.13	0.02	-0.31	-0.01

30 1 0.00 -0.00 -0.00 -0.28 0.05 -0.19 -0.03 -0.14 0.23<br>Zero-point correction= 0.234341 (Hartree/Particle)<br>Thermal correction to Energy= 0.249401<br>Thermal correction to Enthalpy= 0.250346<br>Thermal correction to Gibbs Free Energy= 0.191220<br>Sum of electronic and zero-point Energies= -836.945848<br>Sum of electronic and thermal Energies= -836.930788<br>Sum of electronic and thermal Enthalpies= -836.929844<br>Sum of electronic and thermal Free Energies= -836.988970<br>E (Thermal) CV S<br>KCal/Mol Cal/Mol-Kelvin Cal/Mol-Kelvin<br>Total 156.502 57.334 124.441<br>Electronic 0.000 0.000 1.377<br>Translational 0.889 2.981 42.291<br>Rotational 0.889 2.981 32.526<br>Vibrational 154.724 51.373 48.247<br>HF=-837.1801897	
**Inter2-N3-H20-HAT- Water**	**$C_{11}H_{12}N_2O_2$ + HOO$^{\bullet}$- Water**
0 2<br>O -1.18099600 0.49845200 -1.49958300<br>O -2.46502300 -1.34288800 -1.58348500<br>N 1.60502800 1.33841400 1.23441300<br>N -3.77516600 -0.76588000 0.57745600<br>C -0.07910100 -0.22857400 1.22990100<br>C -1.44956100 -0.77637400 1.40576400<br>C 1.02913000 -0.82025600 0.55851600<br>C -2.43818400 -0.10647000 0.43237100<br>C 2.04643400 0.16652400 0.57176100	

C	0.37927600	1.08890600	1.61542300
C	1.24018700	-2.05226500	-0.06331000
C	3.26854000	-0.05679300	-0.02423700
C	2.47917900	-2.28040100	-0.66253600
C	3.47383500	-1.30119200	-0.64271800
C	-1.99145100	-0.33123800	-1.03994700
H	-1.78926800	-0.59682400	2.43079500
H	-1.45013300	-1.85545700	1.22804900
H	-2.54760900	0.95354300	0.65453200
H	-0.21325400	1.82426200	2.14665100
H	1.30616900	2.68383600	-0.19241300
H	0.46732500	-2.81266500	-0.08454300
H	4.04199300	0.70194500	-0.01910200
H	4.42647100	-1.50386900	-1.11737100
H	-3.76522600	-1.56223500	-0.08178700
H	-4.53442100	-0.14330600	0.29460500
O	0.85822500	3.00873000	-0.99514300
O	-0.51540100	2.93306200	-0.62943800
H	-0.77461100	2.03182700	-0.94224400
H	-3.97362200	-1.09141400	1.52549500
H	2.67301600	-3.22742800	-1.15046600

**Anion (Adibatic) - Water**	**$[C_{11}H_{12}N_2O_2]^-$ - Water**

-1 2			
O	1.58767700	-0.66649200	1.92293700
O	2.63693500	-1.83364400	0.30940300
N	-1.76930400	2.01534200	0.46192000
N	3.73667700	0.27813300	-0.69713900
C	-0.01249200	0.99491300	-0.47673400

C	1.34797200	0.73055000	-1.04430100	
C	-1.08493100	0.06315800	-0.40760800	
C	2.43146600	0.50086200	0.01822000	
C	-2.18273300	0.73644300	0.18452200	
C	-0.44989700	2.19922700	0.05960400	
C	-1.24080800	-1.31434000	-0.80940100	
C	-3.44204700	0.13352200	0.40464500	
C	-2.50151400	-1.91572900	-0.58543000	
C	-3.56477500	-1.23593300	-0.00570300	
C	2.18800400	-0.79154500	0.84731800	
H	1.66575200	1.57526000	-1.66708200	
H	1.31265400	-0.15356000	-1.69133200	
H	2.53888700	1.36998000	0.66437500	
H	0.05035200	3.14575300	0.18674400	
H	-2.34881300	2.72774800	0.87487900	
H	-0.41962300	-1.87354800	-1.24274700	
H	-4.26305900	0.67770900	0.85544800	
H	-4.50891300	-1.75130300	0.14035800	
H	3.75043100	-0.73209700	-0.91447900	
H	4.54197900	0.47697100	-0.10198400	
H	-2.64539600	-2.95358300	-0.87587500	
H	3.82777800	0.83065600	-1.55129100	
**Anion (Vertical) - Water**				**$[C_{11}H_{12}N_2O_2]^-$ - Water**
-1 2				
O	1.33267600	-0.89124100	1.78425000	
O	2.39766200	-1.85467400	0.04804800	
N	-1.87172300	2.02177800	0.42903900	
N	3.73526000	0.30268900	-0.48735500	

C	0.01289400	1.13579500	-0.41486900	
C	1.41692900	0.98174700	-0.90951000	
C	-1.00221000	0.11638200	-0.38315200	
C	2.39793300	0.50180200	0.16896400	
C	-2.17046400	0.70985500	0.14972700	
C	-0.56428100	2.27184600	0.08378800	
C	-1.02938400	-1.23649600	-0.75725900	
C	-3.36094500	-0.00633900	0.31428200	
C	-2.20504500	-1.94812900	-0.59784600	
C	-3.36016600	-1.33690400	-0.06669100	
C	1.99872700	-0.88861000	0.74166500	
H	1.77403100	1.93759200	-1.30420700	
H	1.43653100	0.25891900	-1.73306500	
H	2.50785400	1.23955600	0.96109700	
H	-0.14147300	3.25640100	0.21600800	
H	-2.51175300	2.70266700	0.80632100	
H	-0.13767900	-1.71532800	-1.14834400	
H	-4.24737000	0.46366900	0.72382800	
H	-4.26516300	-1.92209400	0.04667200	
H	3.69610100	-0.64675200	-0.89324700	
H	4.49844200	0.31581700	0.19101200	
H	-2.24374100	-2.99361100	-0.88016000	
H	3.94283500	0.99352600	-1.21068300	
**Cation (Vertical) - Water**				**$[C_{11}H_{12}N_2O_2]^{+\bullet}$ - Water**
1 2				
O	1.33267600	-0.89124100	1.78425000	
O	2.39766200	-1.85467400	0.04804800	
N	-1.87172300	2.02177800	0.42903900	

N	3.73526000	0.30268900	-0.48735500
C	0.01289400	1.13579500	-0.41486900
C	1.41692900	0.98174700	-0.90951000
C	-1.00221000	0.11638200	-0.38315200
C	2.39793300	0.50180200	0.16896400
C	-2.17046400	0.70985500	0.14972700
C	-0.56428100	2.27184600	0.08378800
C	-1.02938400	-1.23649600	-0.75725900
C	-3.36094500	-0.00633900	0.31428200
C	-2.20504500	-1.94812900	-0.59784600
C	-3.36016600	-1.33690400	-0.06669100
C	1.99872700	-0.88861000	0.74166500
H	1.77403100	1.93759200	-1.30420700
H	1.43653100	0.25891900	-1.73306500
H	2.50785400	1.23955600	0.96109700
H	-0.14147300	3.25640100	0.21600800
H	-2.51175300	2.70266700	0.80632100
H	-0.13767900	-1.71532800	-1.14834400
H	-4.24737000	0.46366900	0.72382800
H	-4.26516300	-1.92209400	0.04667200
H	3.69610100	-0.64675200	-0.89324700
H	4.49844200	0.31581700	0.19101200
H	-2.24374100	-2.99361100	-0.88016000
H	3.94283500	0.99352600	-1.21068300

Table S5. Optimized structures of HLP forms in water using M06-2X/6-311++g(d,p)

**Stable Neutral form - Water**	**$C_{11}H_{12}N_2O_3$ - Water**
0 1	

O	-2.52942900	-2.71600500	-0.54976600
O	1.40713000	-0.80584000	1.85196500
O	2.26249600	-2.02739800	0.16318900
N	-1.30653400	2.56370900	0.29984800
N	3.91453300	-0.14337500	-0.52012300
C	0.38029000	1.29243600	-0.46834700
C	1.72427000	0.86090700	-0.96480400
C	-0.78624000	0.46531700	-0.32311900
C	2.63715400	0.30198300	0.13503400
C	-1.82467100	1.29453700	0.15781900
C	0.01097200	2.55436200	-0.08163400
C	-1.03365400	-0.89598400	-0.55948300
C	-3.10554700	0.79738700	0.40414000
C	-2.30322700	-1.38252300	-0.31549800
C	-3.33360700	-0.54589700	0.16117000
C	2.03859800	-0.97068800	0.80101500
H	2.22948000	1.70866500	-1.43734600
H	1.60096800	0.08589700	-1.73008900
H	2.87593900	1.06191300	0.87605300
H	0.59597300	3.46151900	-0.05654200
H	-1.81523100	3.37585600	0.61114500
H	-0.25103100	-1.56491500	-0.90116000
H	-3.89921600	1.43626300	0.77235200
H	-4.31710600	-0.96696000	0.34134900
H	3.72164300	-1.09792200	-0.86515400
H	4.68532200	-0.20554200	0.14678600
H	-3.43949500	-2.93636500	-0.32813900
H	4.20915100	0.46217700	-1.28818300

Cation - Water	$[C_{11}H_{12}N_2O_3]^{\bullet+}$ - Water		
1 2			
O	-2.42681500	-2.66740000	-0.54119900
O	1.22913900	-0.76185600	1.78089600
O	2.14014200	-2.02078500	0.14851300
N	-1.32390200	2.51724400	0.27502400
N	3.91572300	-0.21006500	-0.43901600
C	0.43192500	1.31995700	-0.46547700
C	1.78913600	0.90793900	-0.93549900
C	-0.73450100	0.45750200	-0.34014300
C	2.64265100	0.28457000	0.18045600
C	-1.80695300	1.27991700	0.13307800
C	0.02620600	2.55661100	-0.08596300
C	-0.95226100	-0.87239700	-0.56456500
C	-3.11061700	0.77205300	0.37913500
C	-2.26284700	-1.38114400	-0.31523800
C	-3.32666800	-0.55488400	0.15158200
C	1.93828100	-0.96251200	0.78777500
H	2.30830200	1.77848200	-1.34294100
H	1.68331900	0.17448400	-1.74201400
H	2.88860300	1.01646700	0.94640300
H	0.56921700	3.48693500	-0.03760200
H	-1.85372300	3.32016500	0.59268600
H	-0.17780000	-1.55585200	-0.89563400
H	-3.90025500	1.42048600	0.73596500
H	-4.29993900	-0.99754900	0.32343300
H	3.69351900	-1.14736700	-0.81244300
H	4.65509900	-0.32397100	0.25685900

H	-3.32982000	-2.96184800	-0.35241000	
H	4.27487200	0.39396900	-1.18088900	
**Radical Form (N4-H21) - Water**				**$[C_{11}H_{11}N_2O_3]^{\bullet}$ - Water**
0 2				
O	-2.97795800	-2.52590700	-0.53624100	
O	1.42461500	-0.38717400	1.84482300	
O	2.51393300	-1.98312800	0.68287200	
N	-1.06594400	2.59198900	0.33591900	
N	3.92231100	-0.32148700	-0.68465500	
C	0.39037200	1.07858400	-0.59775900	
C	1.65731700	0.53647800	-1.15547900	
C	-0.85329800	0.39069300	-0.39964000	
C	2.69382800	0.25942300	-0.05237100	
C	-1.71884700	1.34841700	0.17916500	
C	0.14969800	2.41665400	-0.11733700	
C	-1.27205900	-0.91073200	-0.63892700	
C	-3.01151600	1.01229500	0.51810700	
C	-2.58856700	-1.24349500	-0.29106000	
C	-3.44430100	-0.30044200	0.27592500	
C	2.15924200	-0.81140700	0.94485100	
H	2.07951900	1.25931000	-1.86172200	
H	1.46047100	-0.39273200	-1.69863300	
H	2.96807500	1.17474400	0.46742300	
H	0.87770700	3.21969900	-0.12138500	
H	-0.62595700	-1.66499100	-1.07402800	
H	-3.68187000	1.73759400	0.96376700	
H	-4.45734900	-0.58941000	0.53372500	
H	3.77262300	-1.34666500	-0.65467100	

H	4.76052100	-0.11778200	-0.13791600
H	-3.89066700	-2.65641900	-0.25782800
H	4.08642400	-0.00599600	-1.64206700
**Anion (N4-H21) - Water**		**$[C_{11}H_{11}N_2O_3]^+$ - Water**	
-1 1			
O	-2.70600700	-2.64388900	-0.55287800
O	1.55909700	-0.87011900	1.88218700
O	2.35196700	-2.02782000	0.12146200
N	-1.32152600	2.63779300	0.35988800
N	3.93117900	-0.07835900	-0.56903600
C	0.35209300	1.26682400	-0.45797300
C	1.69117800	0.83739100	-0.96818600
C	-0.81826500	0.47608400	-0.31736300
C	2.64685000	0.31220100	0.11355400
C	-1.81957100	1.37046100	0.19027400
C	-0.03064700	2.54523600	-0.03435900
C	-1.13097900	-0.87798200	-0.56232300
C	-3.11468400	0.89058800	0.43857000
C	-2.41508700	-1.31363600	-0.30823900
C	-3.40859000	-0.44160400	0.18894100
C	2.12698900	-0.98769500	0.78786800
H	2.18498200	1.68006400	-1.46636700
H	1.57465700	0.04417100	-1.71759900
H	2.87232200	1.07945700	0.85144600
H	0.61286100	3.42078300	-0.01553100
H	-0.38523600	-1.58042600	-0.92274300
H	-3.88256100	1.55526400	0.82271200
H	-4.40617100	-0.82734800	0.37664400

H	3.76356100	-1.02878500	-0.93509500
H	4.71513000	-0.13101800	0.08282100
H	-3.62185200	-2.81620300	-0.31542800
H	4.18854600	0.55541100	-1.32758800

**Radical Form (O1-H27) - Water**	**$[C_{11}H_{11}N_2O_3]^{\bullet}$ - Water**

0 2			
O	-2.51284500	-2.73374800	-0.56037000
O	1.33224600	-0.78255300	1.81915800
O	2.26514300	-1.99468300	0.16428400
N	-1.40565900	2.46597600	0.30290300
N	3.90042700	-0.08610500	-0.48798300
C	0.34550100	1.28544900	-0.46695200
C	1.70577300	0.90030700	-0.95441200
C	-0.79903800	0.40912300	-0.33946800
C	2.61060100	0.34008200	0.15222700
C	-1.87344100	1.20503100	0.14861300
C	-0.07114300	2.51987300	-0.07043800
C	-0.99757600	-0.93471600	-0.58411900
C	-3.16554100	0.67524900	0.39605200
C	-2.29842700	-1.51697400	-0.34754400
C	-3.36348600	-0.64847100	0.15113600
C	2.00780100	-0.94246700	0.79556500
H	2.19333100	1.77182000	-1.40000300
H	1.60976800	0.13961000	-1.73707900
H	2.82923600	1.09507800	0.90418400
H	0.46888800	3.45279700	-0.02435100
H	-1.94511500	3.25535300	0.62751600
H	-0.20112500	-1.58339000	-0.93378300

H	-3.95982100	1.31181100	0.76747800
H	-4.32811000	-1.11164800	0.32052400
H	3.72679800	-1.04458200	-0.83339500
H	4.66341500	-0.13517900	0.18913300
H	4.19687900	0.52219700	-1.25320400

**Anion (O1-H27) - Water**	**$[C_{11}H_{11}N_2O_3]^+$ - Water**

-1 1

O	-2.77398200	-2.73181800	-0.55899700
O	1.55434400	-0.60002800	1.93233500
O	2.43905400	-1.99225100	0.39919600
N	-1.29915900	2.49725200	0.32003000
N	3.89271300	-0.14200700	-0.66412600
C	0.31372300	1.18356800	-0.52529000
C	1.63053300	0.71874500	-1.06336100
C	-0.87028200	0.37974000	-0.34596100
C	2.64181500	0.33079600	0.02401300
C	-1.85955700	1.23591700	0.18073300
C	0.00009700	2.45493400	-0.11304600
C	-1.16387100	-0.97270300	-0.59953200
C	-3.14653400	0.76173700	0.45858800
C	-2.45204200	-1.49863800	-0.33911800
C	-3.42027800	-0.56797800	0.20091600
C	2.15798200	-0.87004200	0.88635600
H	2.08155200	1.50388300	-1.67922500
H	1.47467700	-0.15201100	-1.71039100
H	2.89423300	1.18608900	0.64740600
H	0.61396000	3.34357300	-0.10039700
H	-1.77413000	3.32089500	0.65091500

H	-0.39952100	-1.63796600	-0.99182000	
H	-3.91011800	1.41883800	0.86311600	
H	-4.41246500	-0.95822700	0.40737100	
H	3.73830700	-1.15180300	-0.82663400	
H	4.71884200	-0.04453100	-0.07233300	
H	4.07495800	0.34088200	-1.54541100	
**Inter1-N4-H21-HAT- Water**				**$C_{11}H_{12}N_2O_3$ + HOO$^{\bullet}$- Water**
0 2				
O	-2.50909500	-3.01630000	-0.00119400	
O	1.30097200	-0.44464400	1.67099500	
O	2.39156200	-2.08363400	0.60823500	
N	-1.27665800	2.25700200	-0.86953000	
N	3.97747000	-0.45830000	-0.65140100	
C	0.41371800	0.81003700	-1.16953200	
C	1.77133100	0.25838900	-1.47217600	
C	-0.75598500	0.07022300	-0.78451300	
C	2.65605800	0.11186700	-0.22517300	
C	-1.79614700	1.00923100	-0.60392600	
C	0.04366600	2.13039300	-1.20470600	
C	-1.00768200	-1.29550600	-0.57620000	
C	-3.08157300	0.61802800	-0.22691000	
C	-2.28066300	-1.67762000	-0.20142300	
C	-3.31272100	-0.73145100	-0.02832800	
C	2.06496400	-0.90226900	0.78610200	
H	2.28404900	0.91884200	-2.17731400	
H	1.68202500	-0.72437000	-1.94854900	
H	2.83649500	1.08000600	0.23935100	
H	0.63483700	2.99987900	-1.45030600	

H	-1.77719100	3.12886000	-0.80129800
H	-0.22904900	-2.04293900	-0.68554100
H	-3.87528500	1.34245900	-0.08945000
H	-4.29975300	-1.07179200	0.26671800
H	3.85433400	-1.48209500	-0.67807300
H	4.71742600	-0.25653000	0.02385700
H	-3.42393100	-3.15617200	0.26213100
O	-0.86952600	1.61820100	2.29235100
O	0.35221600	1.85809300	1.89823400
H	0.78824400	0.92896400	1.74185600
H	4.28329500	-0.11768300	-1.56510500

**TS- N4-H21-HAT- Water**	**$C_{11}H_{12}N_2O_3$ + HOO•- Water**

0 2

O	-3.08121000	-2.77143100	0.35386900
O	1.26253600	0.03651900	1.66516300
O	1.92333300	-2.06849200	1.26083500
N	-0.89940800	2.13962000	-0.93571800
N	3.71812900	-1.35887400	-0.48338100
C	0.42090400	0.30739500	-1.28912400
C	1.64569800	-0.51043800	-1.51591400
C	-0.84433300	-0.16868200	-0.77339000
C	2.57460600	-0.40905800	-0.29023100
C	-1.62261800	0.98162200	-0.55067200
C	0.31076100	1.68564000	-1.37184400
C	-1.33388300	-1.43395600	-0.47432600
C	-2.88989900	0.89388600	-0.00979000
C	-2.61882200	-1.52083000	0.06763800
C	-3.38571400	-0.37627600	0.29958200

C	1.85336600	-0.85430200	1.01480400
H	2.18605700	-0.15862400	-2.39854700
H	1.37873700	-1.55939600	-1.67448300
H	2.97642400	0.59812900	-0.19512000
H	1.07006400	2.38018200	-1.70179200
H	-0.68283000	2.79404400	0.05421900
H	-0.75972400	-2.33804400	-0.64229400
H	-3.48762900	1.77849000	0.17488100
H	-4.37938900	-0.47894000	0.72220800
H	3.38763100	-2.27813200	-0.15080500
H	4.52557400	-1.10025400	0.08742700
H	-3.96928900	-2.72109100	0.72275000
O	-0.19080400	2.96543100	1.20134200
O	1.11051600	2.57190000	1.07112400
H	1.13906700	1.60120400	1.33086500
H	4.02888300	-1.42420800	-1.45467700

Frequencies --	-2780.1092	48.6184	56.7087
Red. masses --	1.1799	6.5930	6.5025
Frc consts --	5.3731	0.0092	0.0123
IR Inten --	66495.3737	14.3019	24.0742

Atom	AN	X	Y	Z	X	Y	Z	X	Y	Z
1	8	0.00	0.01	-0.00	0.14	-0.08	0.00	-0.06	0.25	0.06
2	8	0.00	0.00	-0.00	0.29	-0.16	0.14	0.23	0.04	-0.03
3	7	0.03	0.01	-0.03	0.03	0.01	0.05	-0.03	-0.02	-0.13
4	7	-0.00	0.00	-0.00	0.05	-0.01	-0.11	0.03	-0.22	-0.01
5	6	0.01	0.03	0.01	-0.02	0.08	0.08	-0.01	-0.01	0.00
6	6	-0.00	0.00	0.00	-0.04	0.12	0.06	-0.00	-0.03	0.04
7	6	0.00	0.00	0.00	-0.07	0.05	0.02	-0.03	-0.04	0.02

8	6	0.00	-0.00	0.00	0.04	0.01	-0.05	-0.03	-0.06	0.05
9	6	-0.00	0.01	-0.00	-0.05	0.01	-0.00	-0.04	-0.04	-0.07
10	6	-0.05	-0.03	0.01	0.04	0.05	0.09	-0.02	0.00	-0.09
11	6	0.00	-0.00	-0.00	-0.16	0.06	-0.04	-0.02	-0.08	0.13
12	6	0.01	-0.01	-0.01	-0.10	-0.02	-0.09	-0.05	-0.08	-0.08
13	6	-0.01	-0.00	0.00	-0.21	0.04	-0.13	-0.04	-0.12	0.14
14	6	0.00	0.01	0.00	-0.19	-0.00	-0.16	-0.05	-0.12	0.03
15	6	-0.00	-0.00	0.01	0.17	-0.09	0.04	0.06	0.09	0.03
16	1	-0.01	-0.01	-0.00	-0.08	0.23	-0.01	-0.00	-0.04	0.04
17	1	-0.01	-0.00	0.00	-0.06	0.13	0.17	0.03	-0.03	0.04
18	1	-0.00	-0.00	0.00	0.02	0.00	-0.14	-0.16	-0.07	0.11
19	1	-0.01	0.00	-0.01	0.08	0.07	0.11	-0.00	0.02	-0.11
20	1	0.29	-0.40	0.85	0.01	0.02	0.05	0.00	-0.00	-0.10
21	1	-0.00	0.00	0.00	-0.19	0.10	-0.03	-0.01	-0.07	0.22
22	1	0.00	-0.00	-0.00	-0.08	-0.05	-0.12	-0.06	-0.09	-0.15
23	1	0.00	0.00	-0.00	-0.23	-0.02	-0.23	-0.06	-0.15	0.03
24	1	-0.00	0.00	0.00	0.11	-0.05	-0.01	0.15	-0.20	-0.05
25	1	-0.00	-0.00	0.00	0.08	-0.08	-0.23	-0.02	-0.28	0.01
26	8	-0.06	0.01	-0.02	-0.00	0.03	0.06	0.01	0.09	-0.07
27	8	0.04	0.00	-0.01	-0.00	-0.05	0.05	0.01	0.26	-0.02
28	1	-0.05	-0.11	-0.05	0.05	-0.06	0.02	-0.08	0.29	0.03
29	1	0.01	0.00	0.00	-0.04	0.08	-0.13	0.02	-0.31	-0.01
30	1	0.00	-0.00	-0.00	-0.28	0.05	-0.19	-0.03	-0.14	0.23

Zero-point correction= 0.238237 (Hartree/Particle)

Thermal correction to Energy= 0.254608

Thermal correction to Enthalpy= 0.255552

Thermal correction to Gibbs Free Energy= 0.193675

Sum of electronic and zero-point Energies= -912.168837

Sum of electronic and thermal Energies= -912.152466

Sum of electronic and thermal Enthalpies= -912.151522

Sum of electronic and thermal Free Energies= -912.213399

	E (Thermal)	CV	S
	KCal/Mol	Cal/Mol-Kelvin	Cal/Mol-Kelvin
Total	159.769	62.207	130.231
Electronic	0.000	0.000	1.377
Translational	0.889	2.981	42.486
Rotational	0.889	2.981	33.021
Vibrational	157.991	56.245	53.347

HF=-912.407074

**Inter2-N4-H21-HAT- Water**	**$C_{11}H_{12}N_2O_3$ + HOO•- Water**

0 2

O	3.32796500	-2.58203000	-0.65024100
O	-1.37202900	0.12841000	-1.55371400
O	-2.08768000	-1.99744200	-1.42295400
N	0.87938200	2.03749900	1.15894000
N	-3.67500700	-1.54574500	0.57912900
C	-0.33142900	0.09108100	1.29556000
C	-1.51221300	-0.78922200	1.49170900
C	0.94377300	-0.25377000	0.74012300
C	-2.56490900	-0.55746400	0.39086200
C	1.65925000	0.96356000	0.66639300
C	-0.26274000	1.51298300	1.52758100
C	1.50043600	-1.44628100	0.29509600
C	2.93444100	1.00138600	0.14819000
C	2.80078300	-1.40070800	-0.22555900
C	3.50556700	-0.20023400	-0.29882300

C	-1.95687000	-0.83221500	-1.01313700	
H	-1.96891200	-0.58211000	2.46464900	
H	-1.20478200	-1.83868300	1.47346100	
H	-2.98029600	0.44679400	0.45181800	
H	-1.06149200	2.11764200	1.93965400	
H	0.32006000	3.06532600	-0.44916100	
H	0.97256300	-2.39240900	0.33576900	
H	3.48731500	1.93077100	0.08153400	
H	4.50958100	-0.19819000	-0.70891900	
H	-3.38749600	-2.39176000	0.05987300	
H	-4.54915700	-1.21555000	0.16523600	
H	4.22170400	-2.44910700	-0.98384400	
O	-0.13246900	3.15197500	-1.30811500	
O	-1.45948000	2.74064700	-0.99852900	
H	-1.43842700	1.77198800	-1.19416900	
H	-3.85828300	-1.77416600	1.55806800	
**Inter1- O1-H27-HAT- Water**				**$C_{11}H_{12}N_2O_3$ + HOO•- Water**
0 2				
O	-2.49854000	-2.19062300	-1.02327600	
O	1.29618000	-0.92184100	1.24395200	
O	2.44934800	-1.43348100	-0.61220400	
N	-1.47152000	2.93880700	0.63039000	
N	3.94693900	0.70190400	-0.41401500	
C	0.30911200	1.87308500	-0.23081000	
C	1.69475700	1.62576800	-0.74195800	
C	-0.82418600	0.98852300	-0.28646000	
C	2.57646300	0.78413000	0.19433000	
C	-1.92145400	1.69009200	0.26009700	

C	-0.13900100	3.04130700	0.33088500
C	-1.00394300	-0.32821400	-0.74393900
C	-3.19175500	1.11487300	0.35731600
C	-2.25512800	-0.90126100	-0.62252400
C	-3.34510400	-0.18509000	-0.08583500
C	2.07326600	-0.66986400	0.28389500
H	2.18840100	2.58967400	-0.89574500
H	1.65455200	1.12137100	-1.71439200
H	2.66303100	1.24076000	1.17774900
H	0.40133400	3.95471900	0.52959300
H	-2.03181600	3.66692400	1.04419800
H	-0.18057900	-0.90303900	-1.15698400
H	-4.02742700	1.66322200	0.77555600
H	-4.30436300	-0.68370400	-0.01668500
H	3.90944600	-0.03027400	-1.13759100
H	4.65140700	0.41799900	0.26969200
H	-1.66647300	-2.68125200	-0.97536200
H	4.24990000	1.58487400	-0.82968800
O	-0.20448000	-3.46647900	0.10547800
O	0.28315500	-3.16738700	1.27469100
H	0.72119100	-2.18786400	1.21590900

**TS- O1-H27-HAT- Water**	**$C_{11}H_{12}N_2O_3$ + HOO$^{\bullet}$- Water**

0 2

O	-2.67576300	-1.59979100	-1.19919600
O	0.98635700	-1.15150500	1.32164800
O	1.92660100	-2.07712900	-0.49859700
N	-0.57989200	3.08376900	0.58558500
N	4.04377100	-0.54853200	-0.32785700

C	0.92984700	1.66178200	-0.28081200
C	2.22237100	1.05123500	-0.72460400
C	-0.38476800	1.08395900	-0.40416800
C	2.77163000	-0.00282500	0.25235200
C	-1.30068100	2.00569000	0.16152200
C	0.75794500	2.87561800	0.32259400
C	-0.85462300	-0.12722200	-0.90069100
C	-2.67988000	1.73664500	0.24950100
C	-2.22414900	-0.42674200	-0.78664200
C	-3.12340300	0.52079500	-0.20857200
C	1.80889100	-1.20763100	0.38001600
H	2.97192000	1.83853700	-0.84090600
H	2.09178600	0.57477600	-1.70291800
H	2.99413300	0.43410900	1.22326100
H	1.48989300	3.62293200	0.58802700
H	-0.95968000	3.90437100	1.03313500
H	-0.19155600	-0.87101900	-1.33193300
H	-3.36102700	2.45765500	0.68489800
H	-4.16964200	0.24802600	-0.14329600
H	3.76684100	-1.26720900	-1.01319400
H	4.61824700	-1.00768000	0.38160100
H	-2.22113000	-2.38272700	-0.52007500
H	4.61640200	0.16566200	-0.78195900
O	-1.66960100	-2.99755500	0.41942000
O	-1.43832800	-2.01677300	1.33399500
H	-0.46654200	-1.74989600	1.23812300
Frequencies --	-2747.0477	48.9144	67.8879
Red. masses --	1.2195	8.3409	7.3849

Frc consts -- 5.4222 0.0118 0.0201

IR Inten -- 90199.9626 5.3639 8.2400

Atom	AN	X	Y	Z	X	Y	Z	X	Y	Z
1	8	0.00	0.05	-0.00	0.16	-0.10	-0.11	0.04	0.00	-0.08
2	8	0.01	0.00	-0.00	0.01	0.02	-0.02	-0.11	0.32	0.10
3	8	0.00	-0.00	-0.00	-0.07	0.03	-0.08	-0.13	-0.05	0.28
4	7	-0.01	-0.01	-0.00	-0.04	-0.12	0.16	0.05	-0.07	0.10
5	7	-0.00	0.00	-0.00	-0.02	-0.04	-0.12	-0.10	-0.11	-0.09
6	6	0.01	0.00	0.00	0.01	0.01	0.05	0.01	0.02	-0.10
7	6	0.00	-0.00	-0.00	0.03	0.05	0.02	-0.01	-0.01	-0.12
8	6	-0.00	-0.01	-0.01	0.03	-0.01	-0.04	0.01	0.02	-0.07
9	6	0.00	-0.00	-0.00	0.02	-0.01	-0.05	-0.02	0.03	-0.07
10	6	0.01	0.01	0.00	-0.01	-0.09	0.04	0.04	-0.04	0.06
11	6	0.01	-0.01	-0.00	-0.03	-0.06	0.17	0.04	-0.03	0.01
12	6	0.03	0.01	0.00	0.07	0.01	-0.12	0.02	0.04	-0.12
13	6	-0.01	-0.01	-0.00	0.00	-0.14	-0.01	0.05	-0.09	0.14
14	6	-0.03	-0.05	-0.01	0.09	-0.06	-0.13	0.03	-0.01	-0.04
15	6	-0.01	0.02	0.01	0.05	-0.12	-0.10	0.05	-0.07	0.09
16	6	-0.00	0.00	0.01	-0.02	0.01	-0.05	-0.09	0.11	0.13
17	1	-0.01	0.00	-0.00	0.02	0.07	0.09	0.01	-0.03	-0.18
18	1	-0.00	0.00	0.00	0.04	0.12	-0.01	-0.05	-0.06	-0.09
19	1	0.00	-0.00	-0.00	0.07	-0.06	-0.04	0.08	0.11	-0.12
20	1	-0.00	-0.00	0.00	-0.05	-0.07	0.27	0.04	-0.05	0.05
21	1	-0.01	0.00	0.00	-0.06	-0.17	0.24	0.06	-0.11	0.19
22	1	0.00	-0.00	0.00	0.10	0.05	-0.15	0.01	0.08	-0.20
23	1	-0.00	-0.00	-0.00	-0.03	-0.19	0.04	0.06	-0.14	0.25
24	1	0.00	0.00	-0.00	0.06	-0.16	-0.12	0.06	-0.11	0.15
25	1	-0.00	-0.00	0.00	-0.07	-0.01	-0.12	-0.19	-0.19	0.03

26	1	-0.00	0.00	0.00	-0.00	-0.07	-0.15	-0.07	-0.06	-0.08
27	1	0.46	-0.54	0.68	-0.00	-0.01	0.10	0.09	-0.01	-0.11
28	1	0.00	-0.00	-0.00	-0.02	-0.04	-0.12	-0.09	-0.21	-0.23
29	8	-0.02	-0.02	-0.06	-0.13	0.15	0.29	0.13	-0.01	-0.14
30	8	0.01	0.04	0.03	-0.10	0.36	0.05	0.01	-0.05	-0.08
31	1	-0.15	-0.06	0.01	-0.06	0.22	0.05	-0.02	0.08	-0.02

Zero-point correction= 0.239051 (Hartree/Particle)

Thermal correction to Energy= 0.254961

Thermal correction to Enthalpy= 0.255905

Thermal correction to Gibbs Free Energy= 0.195357

Sum of electronic and zero-point Energies= -912.188856

Sum of electronic and thermal Energies= -912.172945

Sum of electronic and thermal Enthalpies= -912.172001

Sum of electronic and thermal Free Energies= -912.232549

	E (Thermal)	CV	S
	KCal/Mol	Cal/Mol-Kelvin	Cal/Mol-Kelvin
Total	159.991	61.385	127.434
Electronic	0.000	0.000	1.377
Translational	0.889	2.981	42.486
Rotational	0.889	2.981	32.989
Vibrational	158.213	55.423	50.582

HF=-912.4279066

**Inter2-O1-H27-HAT-Water**	**$C_{11}H_{12}N_2O_3$ + HOO$^{\bullet}$- Water**

0 2

O	-2.72835300	-1.64554200	-1.14091800
O	1.14038100	-1.09813500	1.30572400
O	2.16103600	-1.89215100	-0.53612200

N	-0.84006400	3.06858600	0.63357300
N	4.07340800	-0.13443300	-0.39540000
C	0.74977400	1.74174500	-0.24287500
C	2.07247600	1.24536400	-0.73694700
C	-0.53403600	1.07746600	-0.34397100
C	2.76216100	0.26014000	0.21923500
C	-1.49756200	1.96174700	0.22148400
C	0.51163700	2.94263500	0.35343300
C	-0.93999000	-0.14969800	-0.82595700
C	-2.87969600	1.64855500	0.29312500
C	-2.33501700	-0.51576300	-0.75086300
C	-3.28055400	0.44257500	-0.19051400
C	1.94625200	-1.05006900	0.35574400
H	2.73420400	2.10245100	-0.89056700
H	1.94393900	0.75081200	-1.70616800
H	2.95534700	0.71624100	1.18756000
H	1.19475500	3.74078200	0.59923100
H	-1.26470200	3.87223000	1.07382400
H	-0.24443600	-0.87081000	-1.24491500
H	-3.58148600	2.35306900	0.72329000
H	-4.32011400	0.14017800	-0.15950000
H	3.85844000	-0.90428900	-1.04871200
H	4.72644900	-0.49575200	0.30228600
H	-1.65726500	-2.90938200	-0.41518400
H	4.53452700	0.63015300	-0.89143900
O	-1.08398700	-3.41758600	0.19173400
O	-1.08138200	-2.59552700	1.35598600
H	-0.22730100	-2.10140700	1.28048600

Anion (Adibatic) - Water				$[C_{11}H_{12}N_2O_3]^-$ - Water
-1 2				
O	-2.83632600	-2.66655400	-0.52637800	
O	1.73828800	-0.55580800	1.99605500	
O	2.46102800	-2.01078500	0.43766100	
N	-1.20215400	2.53507300	0.30911600	
N	3.91343500	-0.21501500	-0.73856800	
C	0.35383000	1.16237700	-0.53541600	
C	1.63993900	0.63292000	-1.09022000	
C	-0.83973100	0.41630200	-0.34327600	
C	2.68969100	0.29285600	-0.02397800	
C	-1.81171600	1.30646600	0.18200800	
C	0.11610000	2.46982900	-0.13243700	
C	-1.19718900	-0.96035700	-0.58493200	
C	-3.13029800	0.91946200	0.47473800	
C	-2.51371800	-1.33465100	-0.28739000	
C	-3.46760900	-0.46051800	0.21710100	
C	2.24499300	-0.86793400	0.90980900	
H	2.08453500	1.36681300	-1.77275000	
H	1.44293200	-0.27465400	-1.67279300	
H	2.96976500	1.17473200	0.54863300	
H	0.75617200	3.33739500	-0.12396400	
H	-1.65814700	3.37043200	0.63669500	
H	-0.48294700	-1.69557200	-0.93511000	
H	-3.85894500	1.61638600	0.86956900	
H	-4.47147800	-0.82370100	0.41650600	
H	3.73672500	-1.22162300	-0.88910100	
H	4.75727100	-0.12902200	-0.17019100	

H	-3.74487800	-2.81321200	-0.24692100
H	4.08033600	0.25629100	-1.62916200
**Anion (Vertical) - Water**		**$[C_{11}H_{12}N_2O_3]^-$ - Water**	
-1 2			
O	-2.52942900	-2.71600500	-0.54976600
O	1.40713000	-0.80584000	1.85196500
O	2.26249600	-2.02739800	0.16318900
N	-1.30653400	2.56370900	0.29984800
N	3.91453300	-0.14337500	-0.52012300
C	0.38029000	1.29243600	-0.46834700
C	1.72427000	0.86090700	-0.96480400
C	-0.78624000	0.46531700	-0.32311900
C	2.63715400	0.30198300	0.13503400
C	-1.82467100	1.29453700	0.15781900
C	0.01097200	2.55436200	-0.08163400
C	-1.03365400	-0.89598400	-0.55948300
C	-3.10554700	0.79738700	0.40414000
C	-2.30322700	-1.38252300	-0.31549800
C	-3.33360700	-0.54589700	0.16117000
C	2.03859800	-0.97068800	0.80101500
H	2.22948000	1.70866500	-1.43734600
H	1.60096800	0.08589700	-1.73008900
H	2.87593900	1.06191300	0.87605300
H	0.59597300	3.46151900	-0.05654200
H	-1.81523100	3.37585600	0.61114500
H	-0.25103100	-1.56491500	-0.90116000
H	-3.89921600	1.43626300	0.77235200
H	-4.31710600	-0.96696000	0.34134900

H	3.72164300	-1.09792200	-0.86515400	
H	4.68532200	-0.20554200	0.14678600	
H	-3.43949500	-2.93636500	-0.32813900	
H	4.20915100	0.46217700	-1.28818300	
**Cation (Vertical) - Water**				**[$C_{11}H_{12}N_2O_3$]$^{+\bullet}$ - Water**
1 2				
O	-2.52942900	-2.71600500	-0.54976600	
O	1.40713000	-0.80584000	1.85196500	
O	2.26249600	-2.02739800	0.16318900	
N	-1.30653400	2.56370900	0.29984800	
N	3.91453300	-0.14337500	-0.52012300	
C	0.38029000	1.29243600	-0.46834700	
C	1.72427000	0.86090700	-0.96480400	
C	-0.78624000	0.46531700	-0.32311900	
C	2.63715400	0.30198300	0.13503400	
C	-1.82467100	1.29453700	0.15781900	
C	0.01097200	2.55436200	-0.08163400	
C	-1.03365400	-0.89598400	-0.55948300	
C	-3.10554700	0.79738700	0.40414000	
C	-2.30322700	-1.38252300	-0.31549800	
C	-3.33360700	-0.54589700	0.16117000	
C	2.03859800	-0.97068800	0.80101500	
H	2.22948000	1.70866500	-1.43734600	
H	1.60096800	0.08589700	-1.73008900	
H	2.87593900	1.06191300	0.87605300	
H	0.59597300	3.46151900	-0.05654200	
H	-1.81523100	3.37585600	0.61114500	
H	-0.25103100	-1.56491500	-0.90116000	

H	-3.89921600	1.43626300	0.77235200
H	-4.31710600	-0.96696000	0.34134900
H	3.72164300	-1.09792200	-0.86515400
H	4.68532200	-0.20554200	0.14678600
H	-3.43949500	-2.93636500	-0.32813900
H	4.20915100	0.46217700	-1.28818300

Table S6. Optimized structure forms of HOO$^{\bullet}$ in gas phase and water using M06-2X/6-311++g(d,p)

**Radical HOO$^{\bullet}$ - Gas**				**[HOO]$^{\bullet}$ - Gas**
0 2				
O	0.05493500	-0.59902000	0.00000000	
H	-0.87895400	-0.87292300	0.00000000	
O	0.05493500	0.70813500	0.00000000	
**HOOH - Gas**				**HOOH - Gas**
0 1				
O	0.00000000	0.71202700	-0.05308300	
H	0.81565400	0.90168500	0.42466700	
O	0.00000000	-0.71202700	-0.05308300	
H	-0.81565400	-0.90168500	0.42466700	
**Anion vertical HOO^{-} - Gas**				**[HOO]$^{-}$ - Gas**
-1 1				
O	0.05493500	-0.59902000	0.00000000	
H	-0.87895400	-0.87292300	0.00000000	
O	0.05493500	0.70813500	0.00000000	
**Anion adibatic HOO^{-} - Gas**				**[HOO]$^{-}$ - Gas**
-1 1				
O	0.05548700	-0.68745900	0.00000000	

H -0.88778700 -0.86327100 0.00000000	
O 0.05548700 0.79536800 0.00000000	
**Anion vertical HOO^- - Water**	**$[HOO]^-$ - Water**
-1 1<br>O 0.05490700 -0.59691700 0.00000000<br>H -0.87852000 -0.88050600 0.00000000<br>O 0.05490700 0.70698000 0.00000000	
**Anion adibatic HOO^- - Water**	**$[HOO]^-$ - Water**
-1 1<br>O 0.05512900 -0.67541500 0.00000000<br>H -0.88206200 -0.89722400 0.00000000<br>O 0.05512900 0.78756800 0.00000000	
**Radical $HOO^{\bullet}$ - Water**	**$[HOO]^{\bullet}$ - Water**
0 2<br>O 0.05496000 -0.59539000 0.00000000<br>H -0.87935400 -0.88486300 0.00000000<br>O 0.05496000 0.70599800 0.00000000	
**HOOH - Water**	**HOOH - Water**
0 1<br>O 0.00000000 0.70963200 -0.06657900<br>H 0.72945600 0.92579700 0.53263500<br>O 0.00000000 -0.70963200 -0.06657900<br>H -0.72945600 -0.92579700 0.53263500	

Table S7 SOMO at transition states of LP and HLP from N-H and O-H bonds

For the N3-H20 and O1-H20 transfers, the SOMO at the TS is characterized by 2p–π orbitals that are nearly orthogonal to the N3-donor···H···O-acceptor axis and O1-donor···H···O-acceptor axis (as can be seen in Fig. S2a and Fig. S2b). Based on Mayer's framework, this suggests a PCET mechanism. Here, the proton is transferred between lone-pair orbitals on nitrogen and oxygen in σ symmetry, while an electron is simultaneously transferred from a doubly occupied 2p-π orbital on the nitrogen (LP) or oxygen (HLP) to the singly occupied 2p-π orbital on the oxygen of the HOO$^{•}$ radical. In contrast, Fig. S2c also illustrates the SOMO corresponding to H transfer from the N4 atom in HLP. At this TS, the SOMO exhibits a node at the migrating hydrogen atom and is predominantly localized along the N4-donor···H···acceptor vector. This SOMO structure aligns with a HAT transition state, distinguishing it mechanistically from PCET. However, further investigations are required to provide a more precise understanding of the mechanisms underlying HAT and PCET.

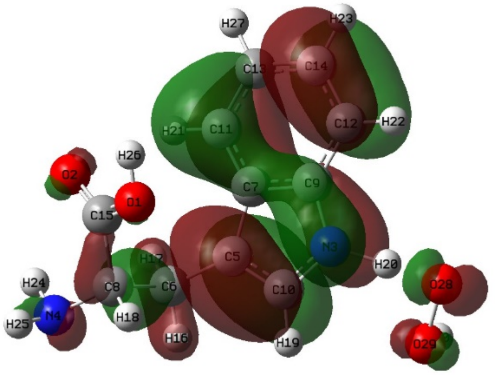

(a) SOMO of N3-H20-LP (PCET)

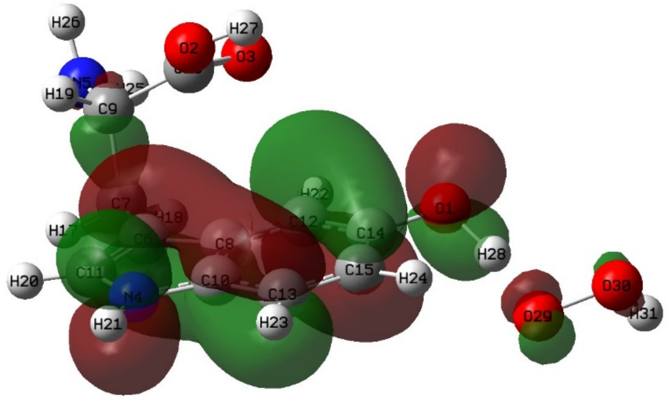

(b) SOMO of O1-H27-HLP (PCET)

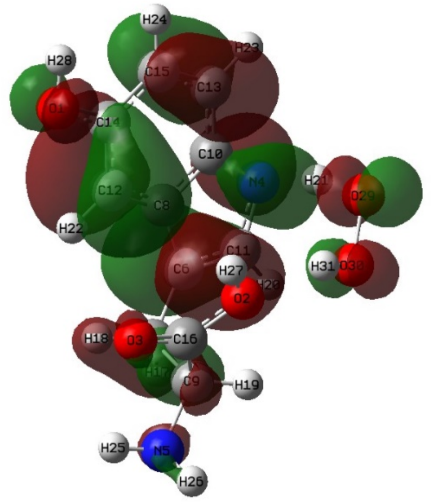

(c) SOMO of N4-H21-LP (HAT)

Fig. S2. SOMO of TSs from N-H and O-H bonds in LP and HLP molecules.
